# Dual pH- and Temperature-Responsive Performance and Cytotoxicity of N-Isopropylacrylamide and Acrylic Acid Functionalized Bimodal Mesoporous Silicas with Core–Shell Structure and Fluorescent Feature for Hela Cell

**DOI:** 10.3390/pharmaceutics17020206

**Published:** 2025-02-06

**Authors:** Huijie Ge, Xiaoli Wang, Shiyang Bai, Yuhua Bi, Fei Liu, Jihong Sun, Wenliang Fu, Donggang Xu

**Affiliations:** 1 Beijing Key Laboratory for Green Catalysis and Separation, Institute of Matter Science, Beijing University of Technology, Beijing 100124, China; gehuijie@emails.bjut.edu.cn (H.G.); wangxiaoli@bjut.edu.cn (X.W.); sybai@bjut.edu.cn (S.B.); biyuhua@emails.bjut.edu.cn (Y.B.); liufei@emails.bjut.edu.cn (F.L.); 2 Beijing Institute of Basic Medical Sciences, Beijing 100850, China; xudg@bmi.ac.cn

**Keywords:** Dual pH-and temperature-responsive carrier, bimodal mesoporous SiO_2_, fractal feature, cytotoxicity, HeLa cell, fluorescent imaging

## Abstract

**Background:** Polymer-coated mesoporous silica nanoparticles have attracted immense research interest in stimuli-responsive drug delivery systems due to their drug-releasing ability on demand at specific sites in response to external or internal signals. However, the relationships between the coated-copolymer encapsulation and drug delivery performance in the hybrid nanocomposites was rarely reported. Therefore, the main objectives of the present work are to explore the cell uptake, cellular internalization, cytotoxicity, and hemolysis performance of the fluorescent hybrid materials with different polymer-encapsulated amounts. **Methods:** Using (2-(2-aminoethyl)-6-(dimethylamino)-1H-benzo[de]isoquinoline-1,3(2H)-dione)-doped poly[(N-isopropylacrylamide)-co-(acrylic acid)] (PAN) as a shell and bimodal mesoporous silicas (BMMs) as a core, the dual pH- and temperature-responsive mesoporous PAN@M-BMMs with the fluorescent performances were synthesized via a radical polymerization approach. The effects of the PAN-coated thicknesses on their physicochemical properties and structural features were demonstrated via XRD and SAXS patterns, SEM and TEM images, FT-IR spectra, and TG analysis. Their mass fractal (*D_m_*) evolutions were elucidated on the basis of the SAXS patterns and fluorescence spectra. **Results:** The *D_m_* values increased from 2.74 to 2.87 with an increase of the PAN-coated amount from 17 to 26.5% along with the particle size from 76.1 to 85.6 nm and blue-shifting of their fluorescent emission wavelength from 470 to 444 nm. Meanwhile, the PAN@M-BMMs exhibited a high ibuprofen (IBU) loading capacity (13.8%) and strong dual pH-/temperature-responsive drug-releasing performances (83.1%) at pH 7.4 and 25 °C, as comparison with that (17.9%) at pH 2.0 and 37 °C. The simulated results confirmed that the adsorption energy decreased from −67.18 kJ/mol for pure BMMs to −116.76 kJ/mol for PAN@M-BMMs, indicating the PAN-grafting on the surfaces of the BMMs core was beneficial to improve its IBU-adsorption capacity. Its uptake in the HeLa cell line was performed via microplate readers, confocal microscopy, flow cytometry, and ICP measurement, showing a low cytotoxicity at a concentration up to 100 µg/mL. Specially, P_0.2_AN@M-BMMs had a superior cellular uptake and fluorescence properties via the time-dependent uptake experiments, and exhibited the highest silicon content via the cellular internalization analysis, as compared to other carriers. Hemolysis tests confirmed the hemolysis rates below 5%. **Conclusions:** These demonstrations verified that PAN@M-BMMs should be a promising biomedical application prospect.

## 1. Introduction

Recently, new therapeutic drugs for treatment of severe diseases have received considerable attention [1]; however, their efficacy is often reduced due to their nonspecific biodistribution and/or rapid clearance in the body [2], in particular, poor water solubility and low cell uptake [3]. The low solubility of most anti-inflammatory drugs results in low bioavailability and even toxic side effects, limiting their wider applications [4]. Ibuprofen (IBU) is one of the most effectively nonsteroidal anti-inflammatory drugs, which is often used in clinical practice for anti-inflammatory, analgesic, and antipyretic therapy [5]. However, due to its poor water solubility and short biological half-life (2 h), its oral absorption and therapeutic effects are greatly limited [6]. Therefore, IBU was initially selected as the drug model to overcome the shortcomings of this type of drug. Drug delivery systems (DDSs) have received tremendous attention to address these deficiencies [7], because of their unique and broad applicability for improving therapeutic effectiveness and/or reduced toxicity [8]. Particularly, the stimuli-responsive DDSs have attracted an immense research interest due to their drug-releasing ability on demand at specific sites in response to external or internal signals [9,10]. Various nanocarriers, such as liposomes [11] and functional porous nanoparticles [12], have been widely investigated. Especially, the mesoporous silica nanoparticles (MSNs) were proposed as a good candidate for biomedical applications [13] with a large surface area [14], controllable porous structure [15], suitable mechanical strength [16], and great biocompatibility [17]. More importantly, the attachment of the functional groups or various nanovalves was allowed to be modified on the mesoporous surfaces of MSNs by the abundant silanol groups for responsive targeting or on-demand drug release, which can be facilely tuned to maximize the cellular uptake [18].

Currently, several research results indicated a good cytotoxicity to A357, A549, Hep2, MCF-7, and HeLa cells by polymer-coated MSNs with various polymers, such as polyethylene glycol (PEG) [19], poly[(N-isopropylacrylamide)-co-(methacrylic acid)](P(NIPAM-co-AA)) [20], and poly(acrylic acid) (PAA) [21]. For example, Palanikumar et al. [22] synthesized the polymer-gatekeeper MSNs modified with disulfide cross-linkable polymers to the load drug (CDDP and doxorubicin). The results showed that glutathione (GSH) was highly cytotoxic to KB cells after release. Cytotoxicity (MCF-7, A549, HeLa) of the efficient therapeutic cisplatin-drug delivery system with a Pt (IV) prodrug conjugated on the fluorescent MSNs surface was investigated by Ahn et al. [23]. The IC50 value of the prodrug-coupled nanoparticles in HeLa cell lines was 63 times lower than that of cisplatin, which was conducive to not only enhancing cell uptake, but also reducing the associated side effects with a high drug effect. Yang et al. [24] prepared the dual pH- and temperature- responsive drug carriers, consisting of a P(NIPAM-co-MAA) copolymer shell and a Fe_3_O_4_/mSiO_2_ core. Its stimuli-responsive performances were attributed to the inclusion of MAA, while the temperature responsiveness was achieved through NIPAM. As reported by Liu et al. [25], using P(NIPAM-co-MAA) as a shell and MSNs modified with double bond as a core, the pH/thermal-triggered nanocomposite was successfully synthesized. Notably, the P(NIPAM-co-MAA) shell exhibited a propensity to undergo network shrinkage at pH (3.5) and temperature (45 °C) conditions. The bimodal mesoporous silicas (BMMs) belong to a unique mesoporous material with hierarchical structure, which is composed of worm-like mesopores of 3 nm and large intergranular pores about 30 nm, which has a high specific surface area and a large pore volume, which is very conducive to improving drug loading. Jin et al. [26,27,28] in our group also prepared the core–shell structured nanocomposites P(NIPAM-co-AA)@BMMs using the dual (thermo- and pH-) responsive P(NIPAM-co-AA) as a shell and BMMs as a core. Using IBU as a model drug, the obtained results revealed that the overall diffusion process was effectively regulated by the coated copolymer shell with the swelling–shrinking behaviors, culminating in a substantial 95% release of IBU at 37 °C and pH 2.0, higher than that (33%) observed at 37 °C and pH 7.4. Specially, the small-angle X-ray scattering (SAXS) patterns were employed to further elucidate the coated thicknesses of the copolymer shell and fractal evolution in the IBU-loading and releasing duration. Meanwhile, we reported a series of luminescent hybrid nanoparticles with a longer fluorescence lifetime (2.2–3.4 ns) by using 1,8-naphenthalic anhydride (NA) derivatives as luminescent probes [29,30,31]. Liu et al. [32] and Wei et al. [33] further explored their fluorescent properties and dual pH-/temperature- responsive performances, suggesting a promising candidate for DDS potential.

However, the relationships between the coated-copolymer encapsulation and drug delivery performance in the hybrid nanocomposites was rarely reported. For these reasons, the main objectives of the present work are to further explore the cell uptake, cellular internalization, cytotoxicity, and hemolysis of the resultant core–shell organic–inorganic hybrid fluorescent nanocomposite. Herein, a series of fluorescent nanoparticles (PAN@M-BMMs) with dual temperature- and pH-responsive performances were synthesized via a radical polymerization approach by using (2-(2-aminoethyl)-6-(dimethylamino)-1H-benzo[de]isoquinoline-1,3(2H)-dione) (AN) as a fluorescent tracer, the uniformly AN-dispersed P(NIPAM-co-AA) as a shell (abbreviated as PAN), and vinyl-grafted BMMs as a core. The temperature- and pH- dual response characteristics under various polymer encapsulation amounts were evaluated by the drug-release performances and the dispersion behaviors of IBU. Meanwhile, the textural parameters and structural features of the fluorescent hybrid nanoparticles PAN@M-BMMs were extensively investigated via various characterizations, such as SAXS and X-ray diffraction (XRD) patterns, fluorescent and Fourier-transform infrared (FT-IR) spectra, dynamic light scattering (DLS), and thermalgravimetric analysis (TGA) measurements, in which the Visual Molecular Dynamics (VMD) program using the Gauss View software 6.0.16 was employed for illustrating the molecular structures of the PAN-coated shell. The (101) surface energy of the SiO_2_ core was calculated at the PBE0-D3/DZVP-MOLOPT-SR-GTH level, integrating Auxiliary Density Matrix Methods (ADMM). Specially, the cytotoxicity and cellular internalization of these nanocomposites was tested in vitro for the HeLa cancer cells. Their uptake process and distributions in the cell were performed by fluorescence imaging.

## 2. Materials and Methods

### 2.1. Materials

Cetyltrimethylammonium bromide (CTAB, A.R.), potassium persulfate (KPS, A.R.), N,N′-methylene bisacrylamide (BIS, A.R.), tetraethyl orthosilicate (TEOS, A.R.), sodium dodecyl sulfate (SDS, A.R.), 3-methacryloxypropyltri-methoxysilane (MPS, A.R.), acrylic acid (AA, A.R.), N-isopropylacrylamide (NIPAM, 98%), and IBU (AR) were supplied by Aladdin Chemistry Co., Ltd. (Shanghai, China) AN was obtained from Shanghai Haohong Bio-Pharm Technology Co., Ltd. (Shanghai, China) Ammonium nitrate (NH_4_NO_3_, A.R.), dimethyl sulfoxide (DMSO, A.R.), ammonium hydroxide (NH_4_OH, 25%), and ethanol (EtOH, A.R.) were purchased from Tianjin Fuchen chemical reagents factory. Phosphoric acid, sodium dihydrogen phosphate, and disodium hydrogen phosphate were provided by Beijing chemical works. All experiments were conducted with ultra-pure water (18.2 MΩ·cm^−1^).

Phosphate-buffered saline (PBS), fetal bovine serum (FBS), glutamine, Dulbecco’s modified Eagle’s medium (DMEM), 0.25% trypsin/EDTA solution, and penicillin/streptomycin were provided by HyClone. Cell Counting Kit-8 (CCK-8) was obtained from Dojindo Molecular Technologies. Human cervical cancer cells (HeLa) were supplied by Saiqi Biological Engineering Co., Ltd. (Zhuhai, China).

### 2.2. Preparation of PxAN@M-BMMs

#### 2.2.1. Synthesis of t-BMMs and Surface Modification

In a 1000 mL beaker, 10.45 g of CTAB was introduced into 416 mL of deionized water, and the mixture was subsequently stirred continuously in a water bath at 40 °C until the CTAB was completely dissolved. Upon removal from the water bath and when the solution had returned to room temperature, 32 mL of TEOS was added dropwise and slowly. Thereafter, 9.6 mL of 25% NH_4_OH was swiftly incorporated, and stirring was maintained until the mixed solution transformed into a gel-like solid. The mixture was then allowed to stand undisturbed at room temperature for 30 min. Through a Buchner funnel, the mixture was filtered, and the resultant solid was thoroughly washed with deionized water until the residual NH_4_OH was removed, ensuring a neutral pH of the eluent. Subsequently, the product was dried overnight in an oven set at 120 °C. The dried material was then ground in an agate mortar to yield a solid powder, designated as t-BMMs.

Subsequently, 1 g of t-BMMs was dispersed in 200 mL of anhydrous ethanol and 1 mL of MPS. The mixture was then stirred at 60 °C for 4 h. Following that, the solid was separated using a Buchner funnel and the resultant sample was thoroughly washed with ethanol. The collected solid was subsequently dried in an oven set at 80 °C overnight. The resultant modified BMMs were designated as M-t-BMMs.

#### 2.2.2. Synthesis of P_x_AN@M-t-BMMs

A series of hybrid nanocomposites P_x_AN@M-t-BMMs were prepared, whereas x represents the mass ratio (wt%) of P(NIPAM-co-AA) with M-t-BMMs, corresponding to 0.2, 0.4, 0.6, and 0.8, respectively.

The detailed synthesis procedure is as follows. In a three-necked flask, 200 mg of M-t-BMMs, 80 mg of NIPAM, 5.6 µL of AA, 14.6 mg of BIS, and 0.3 mg of AN were introduced. The contents were dispersed uniformly in 200 mL of a 0.1 mg/mL sodium dodecyl sulfate aqueous solution. The resulting mixture was then subjected to heating and stirring in an oil bath at 70 °C, while nitrogen gas was bubbled through for 1 h to eliminate any dissolved oxygen. Following this, 108 mg of KPS initiator was swiftly added. The reaction was maintained at 70 °C under a nitrogen atmosphere for an additional 7 h. The reaction mixture was then subjected to centrifugation to remove the supernatant. The resulting solid was washed multiple times with deionized water, followed by centrifugation and drying, yielding the fluorescent hybrid material denoted as P_0.4_AN@M-t-BMMs. Employing the same methodology, P_0.2_AN@M-t-BMMs, P_0.6_AN@M-t-BMMs, and P_0.8_AN@M-t-BMMs were accordingly synthesized. Table S1 in the Electronic Supporting Information (ESI) section summarizes the detailed parameters of the synthesized hybrid BMMs.

In the experimental design, PAN polymers were grafted onto the vinyl-modified surfaces of t-BMMs by radical polymerization, in which a covalent bond was formed between the PAN shell and vinyl-modified surfaces of the BMMs core. Meanwhile, the fluorescent AN was combined via a hydrogen bond between the -NH_2_ and the -CO-NH- (and -COOH) in the obtained PAN polymers or hybrid materials (PAN@M-BMMs).

#### 2.2.3. Removing CTAB

Specifically, 1 g of the obtained t-BMMs, M-t-BMMs, and PxAN@M-t-BMMs were taken and placed into separate 250 mL flasks. Each sample was uniformly dispersed in 200 mL of NH_4_NO_3_-ethanol solution (10 mg/mL). These mixtures were subsequently refluxed for 12 h in an oil bath maintained at 80 °C. This refluxing procedure was repeated thrice for each sample. After refluxing, the samples were washed with ethanol and subjected to centrifugation three times. The resulting precipitates were then dried overnight in a vacuum oven set at 60 °C. Finally, the obtained samples were marked as BMMs, M-BMMs, and P_x_AN@M-BMMs, respectively.

### 2.3. Loading and Releasing of IBU

#### 2.3.1. IBU Loading

Approximately 300 mg of the fluorescent hybrid material was placed in a 50 mL centrifuge tube, and then an ethanol solution containing 20 mg/mL of IBU (30 mL) was added. The mixture was magnetically stirred at room temperature for 48 h. Following that, the solid was separated from the liquid phase using a sintered glass funnel and washed with ethanol. The filtrate was then diluted up to 50 mL in a volumetric flask and then analyzed using high-performance liquid chromatography (HPLC). The obtained solid was dried overnight, and the final product was designated as I/P_x_AN@M-BMMs. The drug-loading content (*LC*%) was calculated using the following formula:

$$LC\%=\frac{m_{1}-c_{1}\times v}{m_{2}+(m_{1}-c_{1}\times v)}\times 100\%$$

where *m*_1_ represents the mass of IBU added (mg), *c*_1_ is the concentration of IBU in the filtrate (mg/mL), *v* is the volume to which the filtrate was made up, and *m*_2_ is the initial mass of the carrier (mg).

Calibration curve setup: An IBU standard solution was prepared using ethanol as a solvent for the drug-loading system.

#### 2.3.2. In Vitro Release of IBU

Approximately 25 mg of the IBU-loaded fluorescent hybrid material was compressed into a disc. This disc was then placed inside a dialysis bag with a cutoff molecular weight of 3500 Da. The bag was immersed in 25 mL of PBS buffer under two different conditions (pH 2.0 at 37 °C and pH 7.4 at 25 °C; the release system at pH 2.0 comprised a mixture of 50% buffer solution and 50% ethanol) in a thermostatic shaker. At specified time intervals (0.5, 1, 1.5, 3, 5, 8, 10, 12, and 24 h), 1.0 mL of the solution was withdrawn. After each sampling, 1.0 mL of fresh PBS solution of the same pH value was added to maintain a constant volume. The IBU-released concentration in the releasing solution was analyzed at a wavelength of 272 nm using a UV–visible spectrophotometer. The calibration standard for IBU and the measured absorbance values for the released IBU in the releasing solution at various time is shown in Figure S1 and Table S2 of the ESI section.

The cumulative drug-release percentage was calculated using the following equation:

$$Cumulative\;Release\;percent\%=\sum_{t=0}^{t}\frac{C_{t}\times V}{M}\times 100\%$$

where *C_t_* (mg/mL) is the concentration at time *t*, *V* is the total volume of the release medium (mL), and *M* is the loading mass of IBU.

**Scheme 1:**
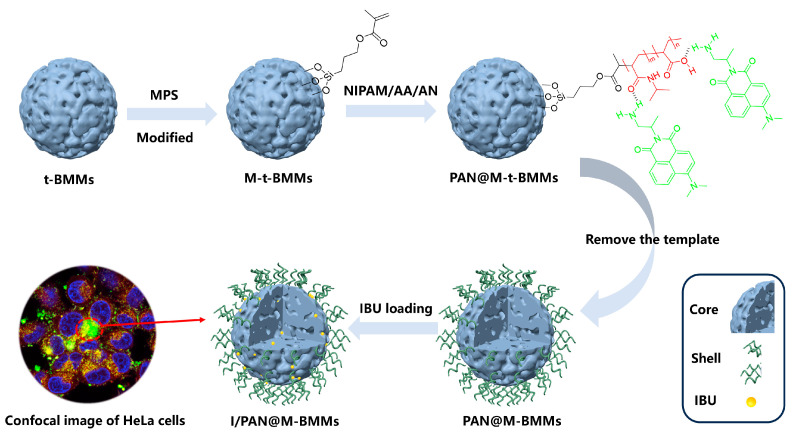
Schematic illustration of the preparation process of the core–shell structured hybrid fluorescent nanoparticles and fluorescent tracer for IBU in HeLa cells.

Scheme 1 illustrates the preparation process of the core–shell structured hybrid fluorescent nanoparticles.

### 2.4. Computational Methods

In the present work, the molecular structures were constructed using the Gauss View 6.0.16 software, while the VMD program was employed for illustrating and rendering of images [34]. PAA and PNIPAM were preoptimized using Gaussian 09 Revision D.01 software [35] at the M06–2X/def2TZVP level prior to grafting. Wave function analysis was conducted through the Multiwfn [36] program. The calculations for structural optimization of inorganic molecules were performed using CP2K [37] at the PBE-D3/DZVP-MOLOPT-SR-GTH. The Perdew–Burke–Ernzerh (PBE) functional, a generalized gradient approximation (GGA) exchange–correlation functional, was selected.

Single-point energy calculations were computed at the PBE0-D3/DZVP-MOLOPT-SR-GTH level with the integration of ADMM. The orbital transformation (OT) method and the independent gradient model of the Hirshfeld partition (IGMH) were used to calculate the self-consistent field (SCF) [38] and the intermolecular interactions, respectively.

The SiO_2_ (101) surface, being the most stable and extensively studied, was chosen as the research substrate [39], which was saturated using OH- groups and employed a 2 × 3 × 1 periodic supercell with five atomic layers and a vacuum thickness of 0.3 nm in the Z-direction for periodic density functional theory (DFT) calculations, simulating the SiO_2_ surfaces. To elucidate the mechanism of the influences of varying thicknesses of organic matter encapsulated in BMMs on the IBU-releasing performances, the effects of different amounts of the encapsulated-P_x_AN shell on the surfaces_(101)_ of SiO_2_ on the IBU loading and releasing behaviors were preliminarily simulated using first principles. Herein, the polymerization degree of PAN was defined as the average of the number of the repeating units of basic building blocks (AA and NIPAM) on the copolymer chains. Therefore, PAN with a polymerization degree of 3 and 5 were grafted onto the surface_(101)_ of SiO_2_, meaning the different encapsulation levels of the PAN shell in the surfaces of the BMMs core.

### 2.5. Cell Culture

The HeLa cell line was employed for this study. HeLa cells were cultured in DMEM supplemented with 10% FBS and were allowed to proliferate under standard conditions at 37 °C with 5% CO_2_ in a humidified atmosphere in the dark.

### 2.6. Cell Viability Assay

The cytotoxicity of the nanohybrid materials in vitro was assessed using the CCK-8 method. First, 96-well flat-bottom tissue culture plates were seeded with HeLa cells at a density of 8 × 10^3^ cells/well (200 µL). The 96-well plates were then incubated for 24 h in a culture chamber. Various materials including BMMs, P_0.2_AN@M-BMMs, P_0.4_AN@M-BMMs, P_0.6_AN@M-BMMs, P_0.8_AN@M-BMMs, P_0.4_AN@M-BMMs-mix, and PAN(NIPAM-co-AA) were diluted to different concentrations (1, 5, 10, 20, 50, 100, and 200 μg/mL) in DMEM. Among them, the P_0.4_AN shell and the M-BMMs were physically mixed, and the obtained solid was named the P_0.4_AN@M-BMMs-mix. After removal of the medium from the 96-well plates, 200 µL of the sample solution was added to each well. Each concentration was tested in triplicate wells. After 48 h of incubation, the supernatant was aspirated from each well. 100 µL of CCK-8 solution was added to each well subsequently, and the 96-well plate was incubated for another 1 h in the culture chamber. Absorbance at 450 nm (*OD*) was measured using an enzyme-linked immunosorbent assay reader. The cell viability percentage was calculated using the following formula:

$$Cell\;Viability(\%)=\frac{{OD}_{Sample}-{OD}_{Blank}}{{OD}_{Control}-{OD}_{Blank}}\times 100\%$$

where *OD_Sample_* and *OD_Control_* are the absorbance values at 450 nm for the sample and control medium, respectively; and *OD_Blank_* represents the absorbance value of the blank medium.

### 2.7. Cell Uptake Study

1 × 10^5^ cells were cultured in glass-bottom dishes, and after 24 h of incubation, fluorescent hybrid material samples at a concentration of 100 μg/mL were introduced into the dishes, followed by further incubation for 6, 24, and 48 h. The culture medium was removed from the dishes, and the glass-bottom dishes were washed. The cytopainter (cell plasma membrane staining kit-orange fluorescence) was added to the dishes to incubate and stain and fixed with 4% paraformaldehyde. The intracellular distribution of the nanomedicine carriers and the acquisition of fluorescence images were observed using a laser confocal fluorescence microscope.

### 2.8. Flow Cytometry

1 × 10^5^ cells were seeded into a 24-well plate, and following incubation, samples with a concentration of 100 μg/mL were added to respective wells. After 24 h of incubation, the culture medium was discarded, the 24-well plate was washed with PBS, and then digested with trypsin in an incubator for 3 min. The cell suspension was homogenized by pipetting, then centrifuged and resuspended in 0.3 mL of PBS for analysis in a flow cytometer.

### 2.9. Cellular Internalization

The same procedure for fluorescence detection by a microplate reader was repeated. The wells containing cells were thoroughly washed with PBS, followed by trypsin digestion. The total cell count per mL in each well was determined using a cell-counting chamber. Cell suspensions were then digested for 48 h with hydrofluoric acid and 2% nitric acid solution. Inductively Coupled Plasma (ICP) analysis was subsequently performed on the digested cell samples to measure the silicon content.

### 2.10. Hemocompatibility Test of PxAN@M-BMMs

Fresh blood was initially extracted from rabbits and placed into anticoagulant tubes. Following centrifugation at 3000 rpm for 10 min, the supernatant plasma was discarded, and the sediment was washed several times with saline until the supernatant appeared clear, resulting in the collection of red blood cell sediment. The red blood cells were then diluted with saline to prepare a 2% red blood cell suspension. Five concentrations of PxAN@M-BMMs were prepared: 10 μg/mL, 25 μg/mL, 50 μg/mL, 75 μg/mL, 100 μg/mL, and 200 μg/mL. To each concentration, 1.5 mL of the solution was added to centrifuge tubes. Equal volumes of deionized water and saline were used as positive and negative controls, respectively. Subsequently, 1.5 mL of the red blood cell suspension was added to each solution. After incubation at 37 °C for 1 h and subsequent centrifugation, the absorbance of the supernatant from each sample was measured. The hemolysis rate of red blood cells incubated with different concentrations of PxAN@M-BMMs for 1 h was evaluated to assess the biocompatibility of the synthesized carriers in blood. The maximum absorption wavelength of hemoglobin determined using a UV–Vis spectrophotometer was 576 nm, which was used to measure the hemolytic activity of PxAN@M-BMMs. The hemolysis rate (HR) was calculated as follows:

$$HR(\%)=\frac{{OD}_{Sample}-{OD}_{negative}}{{OD}_{positive}-{OD}_{negative}}\times 100\%$$

### 2.11. Characterizations

The crystallographic nature and ordered structure of the PAN@M-BMMs were studied using an XD6 X-ray polycrystal diffractometer (Beijing Purkinje General Instrument Co., Ltd., Beijing, China) using Cu-Kα radiation (λ = 0.154056 nm) with a scanning speed of 1 °/min at 36 kV and 20 mA. The FT-IR spectra of the bare and functionalized samples were acquired using a PerkinElmer Spectrum 100 spectrometer (Perkin Elmer Instruments Co., Ltd., Springfield, IL, USA) via KBr tableting in the spectral range of 4000–400 cm^−1^ at a resolution of 1 cm^−1^.

The DLS measurements of the related samples were performed, in which a 5 mg sample was ultrasonically dispersed into an aqueous solution at room temperature for 0.5 h to determine its particle size in the swollen state at pH 7.0. After standing for 0.5 h, the supernatant was taken for the DLS test. The TGA profiles were determined on a PerkinElmer Simultaneous Thermal Analyzer (STA-8000, PerkinElmer Ltd., Springfield, IL, USA) under an N_2_ atmosphere.

The SAXS patterns were recorded at the Beijing Synchrotron Radiation Facility, in which the two-dimensional Pilatus 1 M-F detector (hybrid pixel, DECTRIS, Baden, Switzerland) with the slit-collimated Kratky compact small-angle system and the incident X-ray wavelength of 0.154 nm was used. The sample-to-detector distance was 1560 mm, which was calibrated with the diffraction ring of a standard sample. The scattering vector magnitude *q* ranged from 0.08 to 3.05 nm^−1^. The one-dimensional data were transferred by two-dimensional SAXS images using the Fit2D software (http://www.esrf.eu/computing/scientific/FIT2D, accessed on 20 January 2025) and further processed use the S program package. The power law (*I*(*q*) = *I*_0_*q* − *α*) was used to calculate the microstructure information with the mass fractal values (*D_m_*, 1 < *α* < 3) or surface fractal values (*D_s_*, 2 < 6 − *α* < 3).

## 3. Results and Discussion

### 3.1. Structural and Textural Characterizations

**Figure 1:**
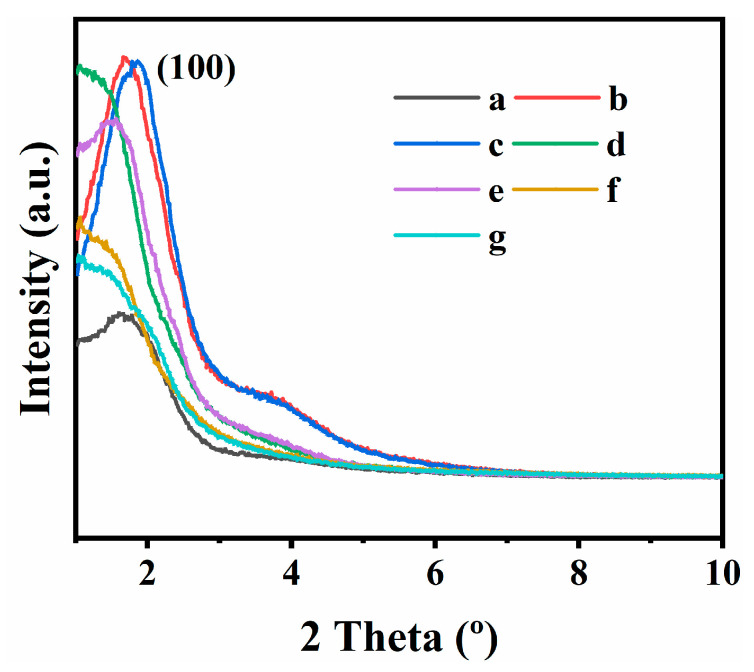
XRD patterns of t-BMMs (a), BMMs (b), M-BMMs (c), P_0.2_AN@M-BMMs (d), P_0.4_AN@M-BMMs (e), P_0.6_AN@M-BMMs (f), and P_0.8_AN@M-BMMs (g).

A key feature of the resultant PAN@M-BMMs for dual pH-and temperature-responses was the conjugating AA and NIPAM onto the amine-functionalized surfaces of M-t-BMMs. The PxAN@M-BMMs were successfully synthesized by varying P(NIPAM-co-AA) amounts in the PAN@M-BMMs and followed by conjugating fluorescence. As can be seen in Figure 1, the XRD patterns exhibited a wide and intense diffractive peak_(100)_ at 2*θ* around 2° for all samples, indicating a uniform and rich mesoporous structure, being consistent with previous literature [40,41,42].

In detail, the diffractive peak_(100)_ of the BMMs (Figure 1b) shifted slightly toward a larger angle 2*θ* region (1.71°) along with an enhancement in peak intensity, in comparison to that of the t-BMMs (1.62°, as shown in Figure 1a), and the corresponding *d* value decreased from 5.45 nm for t-BMMs to 5.16 nm for BMMs, suggesting an effective removal of the used CTAB. The diffractive peak_(100)_ of the M-BMMs (Figure 1c) shifted slightly toward a larger angle 2*θ* region (1.85°), in comparison to that of the BMMs (1.71°, as shown in Figure 1b), and the corresponding *d* value decreased from 5.16 nm for BMMs to 4.77 nm for M-BMMs, suggesting that the ordered mesopore structures could intact after surface functionalization with vinyl groups by MPS, but the interplanar spacing was somewhat shrunk [29,43]. As shown in Figure 1 from d to g, the diffraction peak_(100)_ of PxAN@M-BMMs gradually decreased in intensity with the increase in P(NIPAM-co-AA)-coated amount, indirectly suggesting the successful coating of P(NIPAM-co-AA) onto the surfaces of M-BMMs [44].

Figure S2 presents the FT-IR spectra of the various samples. As depicted in Figure S2a, the characteristic absorption peaks observed at 1082 and 802 cm^−1^ could be attributed to the vibrations of Si-O-Si in BMMs, and another peak located at 956 cm^−1^ was assigned to the vibration of Si-OH [40]. While the M-BMMs, which underwent modification with vinyl groups (Figure S2b), exhibited the additional distinct peaks at 1711 cm^− 1^ (v-C=O) and 1634 cm^−1^ (v-C=C), corresponding to the characteristic features of 3-(trimethoxysilyl)propyl methacrylate [45]. These results further indicated the successful vinyl-modified surfaces of the BMMs. Figure S2c showed that the noticeable characteristic absorption peaks emerged at 1645, 1547, and 1461 cm^−1^, respectively, which should be assigned to the stretching vibrations of C=O, N-H, and C-N in the amide linkages [45]. Additionally, a shoulder peak that appeared around 1715 cm^−1^ was associated with the C=O stretching vibration of the carboxylic groups in P(NIPAM-co-AA) [45], while the typical stretching vibration peaks of the aromatic ring centered at 786 and 768 cm^−1^ were consistently observed in AN (Figure S2d). Remarkably, the appearances of these characteristic peaks could be observed in PAN@M-BMMs (Figure S2e), demonstrating the successful doping of AN into the PAN@M-BMMs.

**Figure 2:**
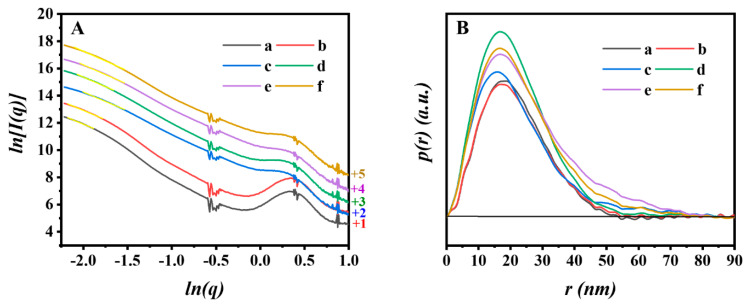
Ln-Ln plots originating from the SAXS patterns (**A**) and PDDF profiles (**B**) of BMMs (a), M-BMMs (b), P_0.2_AN@M-BMMs (c), P_0.4_AN@M-BMMs (d), P_0.6_AN@M-BMMs (e), P_0.8_AN@M-BMMs (f). Yellow lines in Figure 2A: the fitting lines based on the power law, the vertical offset values were presented in the right Y-axis.

The SAXS patterns of BMMs-based samples are displayed in Figure 2, in which each scattering curve was fitted linearly using the least-squares method within the lower scattering vector q range, exhibiting a good linearity with linear correlation coefficients (*R*^2^ > 0.99), implying that all samples possess the typical fractal characteristics. Their fractal dimensional values and related parameters are summarized in Table S3.

### 3.2. Fractal Features

As can be seen in Figure 2A, all samples in the low-*q* region presented the slope values between −2 and −3 (−2.18 < *lnq* < −1.54), indicating the manifestation of the *D_m_* characteristics in values of around 2–3. As demonstrated by Xu et al. [41] and Zhao et al. [46], an increase in the *D_m_* value is indicative of the more densely fractal structures, while its decrease denotes a transition toward more open structures. It should be noteworthy that the fractal structure with a *D_m_* value of 3 represents a dense object. In detail, Figure 2A-a and -b exhibited a slight increase in *D_m_* value from 2.70 for BMMs to 2.71 for M-BMMs, suggesting that the fractal structure of M-BMMs tended to be densified via vinyl-modified surfaces of BMMs. While, the *D_m_* value was 2.74 for P_0.2_AN@M-BMMs (Figure 2A-c), 2.83 for P_0.4_AN@M-BMMs (Figure 2A-d), 2.85 for P_0.6_AN@M-BMMs (Figure 2A-e), and 2.87 for P_0.8_AN@M-BMMs (Figure 2A-f), respectively, exhibiting an increasing tendency with an increasing amount of the coated-PAN. These results implied that the modification with organic functional groups (vinyl groups) and the encapsulation of the coated-PAN onto the surfaces of BMMs tended toward the densification evolution.

As shown in Figure 2B, the PDDF profiles provided insight into the geometric information of the aggregated particles of BMMs-based nanocomposites. As can be seen, the PDDF curves of BMMs (Figure 2B-a), M-BMMs (Figure 2B-b), and PAN@M-BMMs (Figure 2B from c to f) exhibited the symmetrical shapes, indicating the spherical morphology. Meanwhile, the intersection points of the curves with the x-axis represented their maximum particle size (*D_max_*) [47], in which the *D_max_* value increased from 53.4 nm for BMMs (Figure 2B-a) to 54.4 nm for the vinyl-modified M-BMMs (Figure 2B-b), 76.1 nm for P_0.2_AN@M-BMMs (Figure 2B-c), 67.8 nm for P_0.4_AN@M-BMMs (Figure 2B-d), 79.3 nm for P_0.6_AN@M-BMMs (Figure 2B-e), and 75.6 nm for P_0.8_AN@M-BMMs (Figure 2B-f), respectively. These results suggested that the particle size of PAN@M-BMMs should be proportional to the amount of the coated-PAN encapsulation. Aforementioned, we could speculate the thickness of the coated-PAN shell in the obtained PAN@M-BMMs, showing 10.8 nm for P_0.2_AN@M-BMMs, 7.7 nm for P_0.4_AN@M-BMMs, 12.5 nm for P_0.6_AN@M-BMMs, and 10.5 nm for P_0.8_AN@M-BMMs, respectively.

The particle size profiles obtained via the DLS measurements were presented in Figure S3 of the ESI section, showing the narrow and unimodal distributions. As can be seen, an average hydrodynamic diameter (*D_h_*) of BMMs was approximately 131 nm, larger than the size of about 30–50 nm measured on the SEM image of our previous report [48]. The main reason is due to the presences of the hydration layer thickness on the BMMs surfaces in the DLS measurements. Meanwhile, the *D_h_* value gradually increased with the increasing amount of the coated-PAN shell, showing 155 nm for P_0.2_AN@M-BMMs, 179 nm for P_0.4_AN@M-BMMs, 205 nm for P_0.6_AN@M-BMMs, and 297 nm for P_0.8_AN@M-BMMs, respectively, similar to variation tendencies deriving from the PDDF demonstrations (Figure 2B).

The TGA curves of all related samples were depicted in Figure S4. It was observed that the weight losses of all samples below 200 °C were attributed to the moisture absorbed in the samples or residual solvents in the mesoporous channels [43,48]. Specifically, the BMMs sample (Figure S4a) showed a weight loss of approximately 18.3 wt% between 200 and 800 °C, owing to the decomposition of bound water between silanol groups and organic components remaining in the channels. In contrast, the M-BMMs sample (Figure S4b) exhibited a weight loss of 17.6 wt%, potentially due to the decomposition of the MPS-grafted surfaces of the BMMs. Consequently, using the M-BMMs sample as a reference, the PAN-encapsulated amount was roughly calculated. As indicated in Figure S4c–f, the weight loss of P_x_AN@M-BMMs in the range of 200–800 °C noticeably increased with the increasing PAN-grafted amounts (0.2, 0.4, 0.6, and 0.8), which can be attributed to the decomposition of the vinyl-modified and PAN-coated surfaces. The PAN-encapsulated amounts were estimated to be 17 wt% for P_0.2_AN@M-BMMs, 21.5 wt% for P_0.4_AN@M-BMMs, 24.6 wt% for P_0.6_AN@M-BMMs, and 26.5 wt% for P_0.8_AN@M-BMMs. The polymerization conversion rate was approximately 68%, 46%, 36%, and 30%, respectively, and the conversion rate gradually decreased with the increase in concentration. The fluorescent emission spectra of the fluorescent hybrid materials with different polymer-encapsulated amounts are shown in Figure S5 of the ESI section. As can be seen, the obvious blue shift of the fluorescent peaks appeared with the increasing polymer-encapsulated amounts, showing 470, 462, 458, and 444 nm for P_0.2_AN@M-BMMs, P_0.4_AN@M-BMMs, P_0.6_AN@M-BMMs, and for P_0.8_AN@M-BMMs, respectively.

In order to investigate the temperature- and pH-sensitive performances of the core–shell structured PAN@M-BMMs, their hydrated particle size (*D_h_*) was determined via DLS measurements in PBS solution (pH 2.0 and 7.4) at 25 and 37 °C. As shown in Figure S6 of the ESI section, the particle size distribution profiles of P_0.2_AN@M-BMMs, P_0.4_AN@M-BMMs, P_0.6_AN@M-BMMs, and P_0.8_AN@M-BMMs at different temperatures and pH values showed the single peaks, corresponding to their specific mean *D_h_* values, which are summarized in Figure S7.

Taking P_0.2_AN@M-BMMs (Figure S7a) as an example, the average *D_h_* value was around 299.9 nm at pH 7.4/25 °C, but decreased to 278.1 nm at pH 2.0/25 °C, obviously showing the pH-sensitive performances. This phenomenon could be well interpreted as follows. When the pH value is higher than the pKa (4.75) of PAA, the -COOH groups existing in the PAN copolymers are deprotonated, leading to the swollen occurrences due to their electrostatic repulsions among the -COO- groups. While, when pH value below its pK_a_, the intramolecular hydrogen bonding forces among the -COOH groups are enhanced due to the protonation of the -COOH groups, showing the shrunk networks. Similarly, the *D_h_* value of P_0.2_AN@M-BMMs was around 210.8 nm at pH 2.0/37 °C. This is due to the formation of the hydrogen bonds between the amide groups located on the PNIPAM chains and the water molecules, showing the swelling state when the temperature is lower than the critical solution temperature (LCST, around 32 °C) of PNIPAM. As the temperature rises above LCST, the hydrogen bonds are broken, leading to the enhanced hydrophobicity with the swollen states. Obviously, these swollen or shrunk copolymers result in the appearances of the larger or smaller particles of the PAN@BMMs, showing the temperature- and pH-sensitive performances.

The P_0.4_AN@M-BMMs (Figure S7b), P_0.6_AN@M-BMMs (Figure S7c), and P_0.8_AN@M-BMMs (Figure S7d) showed almost the same trends in the particle size, indicating the good dual temperature- and pH-sensitivity properties.

### 3.3. In Vitro Drug Loading and Release

Taking P_0.4_AN@M-BMMs as an example, its IBU-loaded capacity was found to be 13.8%, higher than that (7.9%) of pure P(NIPAM-co-AA), but lower that (23%) of BMMs, indicating an effective loading into the mesoporous channels of the BMMs core.

**Figure 3:**
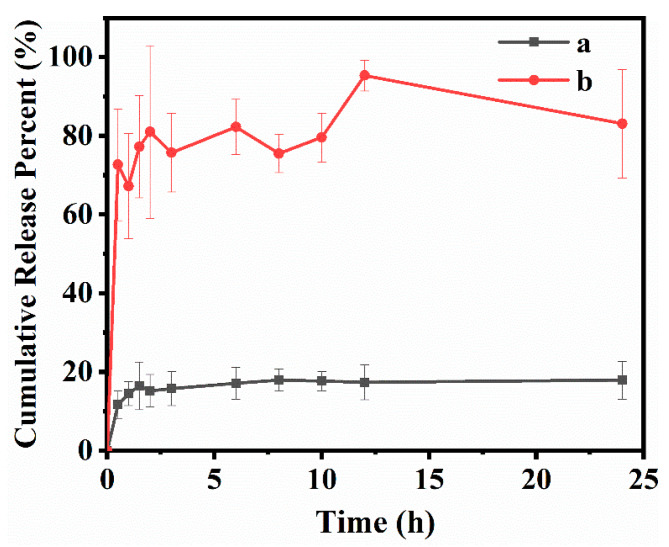
Cumulative IBU-releasing profile from I/P_0.4_AN@M-BMMs in PBS at pH 2.0 and 37 °C (a), and at pH 7.4 and 25 °C (b).

Figure 3 shows its dual pH- and temperature-responsive performances in the IBU-releasing duration. As can be seen, the equilibrium IBU-releasing amount reached approximately 83.1% within 24 h under alkaline conditions (pH 7.4) at 25 °C, higher than that (approximately 17.9%) under acidic conditions (pH 2.0) at 37 °C. These phenomena could be well interpreted as follows. The coated-PAN shell exhibited a swollen state at pH 7.4 and 25 °C due to the electrostatic repulsion of deprotonated carboxylic groups [49], which is conducive to facilitating the opening of network structures and therefore leading to an easier IBU-diffusion from the mesoporous channels of the BMMs core. In contrast, the coated-PAN shell presented a shrunk state at pH 2.0 and 37 °C, due to the hydrogen bonding among the carboxylic groups and the hydrophobic interactions among the -CH(CH_3_)_2_ groups at above the lower critical solution temperature of PNIPAM [50]. These shrunk networks of the shell are beneficial to effectively encapsulate the mesoporous BMMs core, playing a role in hindering the IBU-releasing.

Obviously, the in vitro IBU-releasing performances from P_0.4_AN@M-BMMs demonstrated that the coated-PAN shell actually acted as an effective stimulus-responsive “on–off” switch, exhibiting a pH- and temperature-dependent response, allowing the regulation of drug-releasing behaviors.

The IBU-releasing profiles form P_0.2_AN@M-BMMs, P_0.6_AN@M-BMMs, and P_0.8_AN@M-BMMs are shown in Figure S8, and similar results were also demonstrated in our previous report [51]. As can be seen, their equilibrium cumulative IBU-released percent at pH 7.4/25 °C presented the gradually decreased tendencies with an increasing encapsulated-copolymer amount, showing 92.3, 58.4, and 53.8% for P_0.2_AN@BMMs-7 (Figure S8Ba), P_0.6_AN@BMMs-7 (Figure S8Bb), and P_0.8_AN@BMMs-7 (Figure S8Bc), respectively, but higher than that (44.7% for P_0.2_AN@BMMs-7 (Figure S8Aa), 25.1% for P_0.6_AN@BMMs-7 (Figure S8Ab), and 20.8% P_0.8_AN@BMMs-7 (Figure S8Ac), respectively) at pH 2.0/37 °C.

Obviously, these results imply that the IBU-released performances are strongly related to the encapsulated-copolymer amount, in which the lengths of the IBU diffusion path plays an important role in controlling the drug-released behaviors. Particularly, the increased thickness of the encapsulated-copolymer shell regulated the drug-diffusion behaviors through a longer diffusion path.

As shown in Figure S9A and Table S4, the *D_m_* value of P_0.2_AN@M-BMMs presented the increased trend during the IBU-loading process, showing 2.76 at 1 h (Figure S9Aa), 2.83 at 3 h (Figure S9Ab), 2.85 at 5 h (Figure S9Ac), and 2.86 at 8 h (Figure S9Ad). These results implied its microstructure evolution from loose to dense. However, we noted that when the IBU-loaded time was prolonged to more than 12 h, its fractal characteristics varied from *D_m_* to *D_s_*, and the corresponding *D_s_* value was around 2.99 within 12–48 h (Figure S9Ae,f,g), indicating the occurrences of the rough surfaces. Meanwhile, the PDDF curves, as shown in Figure S9B, exhibited that the *D_max_* value of P_0.2_AN@M-BMMs also presented the increased trend with the extension of IBU-loading time from 81.2 nm at 1 h to 90.2 nm at 48 h, indicating the successful encapsulation of the IBU.

On the other hand, Figure S10 presents the SAXS patterns and PDDF profiles of P_0.2_AN@M-BMMs during the drug-releasing at pH 2.0/37 °C and pH 7.4/25 °C. As shown in Figure S10A and Table S5, the fractal characteristics of P_0.2_AN@M-BMMs underwent a transition from *D_s_* to *D_m_* when IBU was released at pH 2.0/37 °C. In details, the *D_s_* value in the beginning of 1 h was 2.97 (Figure S10Aa). After that, the reverted *D_m_* value was around 2.69 at 3 h (Figure S10Ab), 2.68 at 5 h (Figure S10Ac), and 2.64 at 24 h (Figure S10Ag). These results indicate that the fractal microstructures of P_0.2_AN@M-BMMs happen from the dense structures to the smooth surfaces in the IBU-releasing durations. Correspondingly, its PDDF curves, as shown in Figure S10B, exhibited the symmetric profiles with the estimated *D_max_* values of about 85.8–86.3 nm, suggesting the remaining of the spherical morphologies and sizes during the IBU-releasing process. Similarly, Figure S10C and Table S6 presented the almost same fractal evolutions of P_0.2_AN@M-BMMs at pH 7.4/25 °C as that at pH 2.0/37 °C during the IBU-releasing process. Meanwhile, its *D_max_* values (Figure S10D) were gradually increased in the range of 57.5–87.8 nm at 1–24 h, possible due to the occurrences of the swollen networks at pH 7.4/25 °C in the coated PAN shell.

### 3.4. Simulations for IBU-Adsorption Energy in PAN@M-BMMs

In the present work, the effect mechanism of the coated-PAN shells with different thickness on the IBU-release in PAN@M-BMMs is elucidated by first principles, in which the surfaces_(101)_ of the used SiO_2_ core was assumed to be coated with different amounts of PAN; i.e., the PAN with polymerization degrees of 3 and 5 was grafted onto the surface_(101)_ of SiO_2_, representing different encapsulation levels. The methodology for calculating adsorption energy is detailed as follows [52]:

$$E_{a}=E_{BMMs+IBU}-(E_{BMMs}+E_{IBU})$$

where *E_a_* is the adsorption energy, *E_BMMs_* is the BMMs energy, and *E_IBU_* is the energy of IBU; their IBU-adsorption energies are illustrated in Figure S11. As can be seen, the adsorption energy decreased from −67.18 kJ/mol for pure BMMs (Figure S11a) to −105.07 kJ/mol and −116.76 for PAN@M-BMMs with polymerization degrees of 3 and 5 (Figure S11b,c). These simulated results indicated that the PAN-grafting on the surface_(101)_ of the SiO_2_ core should be beneficial to improve its IBU-adsorption capacity, especially with the increase in the PAN-coated thickness.

To further investigate the mechanism underlying the impact of BMMs grafted with PAA and NIPAM of different polymerization degrees on IBU, the intermolecular interactions between the surfaces_(101)_ of BMMs core and IBU molecules were simulated using the IGMH calculations [53], and the results are shown in Figure S12. As profiled in Figure S12a,b, the green regions represented the presence of the van der Waals forces between IBU and the surface_(101)_ of SiO_2_. Obviously, an area expansion with the increases of the polymerization degree from 3 to 5 implied an enhancement in van der Waals forces. Further observation in conjunction with the changes in adsorption energy associated with different polymerization degree (as shown in Figure S11) suggested that the van der Waals attraction between the oxygen atoms in the IBU molecule and the hydrogen atoms on the surface_(101)_ of SiO_2_ is one of the main factors. Particularly, the hydrogen bonds between the oxygen atoms in IBU and the hydrogen atoms on the surface_(101)_ of SiO_2_ were additionally generated when the polymerization degree increased up to 5 (as shown in Figure S12c), further enhancing the IBU-adsorption capacity. Additionally, van der Waals forces were also observed between the loaded-IBU molecule and the coated-PAN shell.

### 3.5. Cytotoxicity Assessment

**Figure 4:**
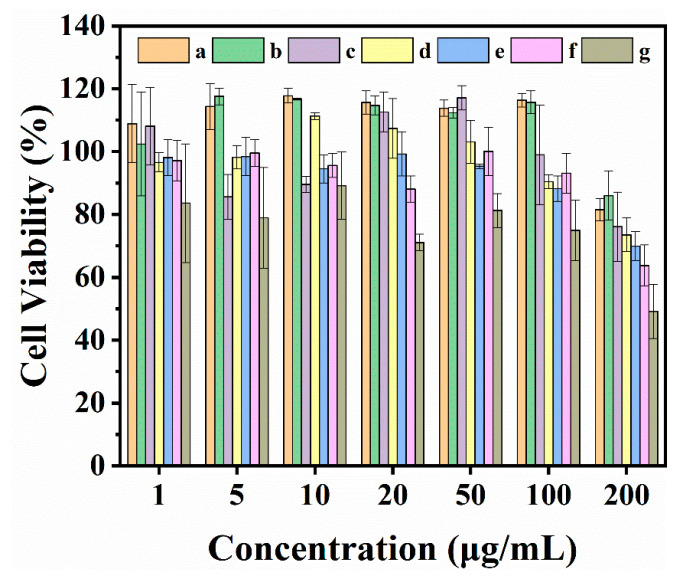
Cell viability to HeLa cells of BMMs (a), P_0.2_AN@M-BMMs (b), P_0.4_AN@M-BMMs (c), P_0.6_AN@M-BMMs (d), P_0.8_AN@M-BMMs (e), P_0.4_AN@M-BMMs-mix (f), and PAN (g).

The cytotoxicity of HeLa cells cultured for 48 h was investigated using the CCK-8 method. As shown in Figure 4 from a to f, all samples showed low cytotoxicity with cell survival rates above 85% at concentrations of the used samples ranging from 1 to 100 μg/mL. However, the general tendencies of the decreased cell viability were observed as the increases of the coated-PAN amount at the same concentrations. However, Figure 4g presented that the pH/temperature dual-responsive fluorescent PAN exhibited a certain level of cytotoxicity, which were, to an extent, dose-dependent. For instance, when cultured with HeLa cells at a concentration of 1 μg/mL, cell viability exceeded 84%. Thus, the polymer-encapsulated PAN@M-BMMs demonstrated very low cytotoxicity. Similar findings were also reported by Kankala et al. [54] and Dong et al. [55].

### 3.6. Cell Uptake Behaviors

**Figure 5:**
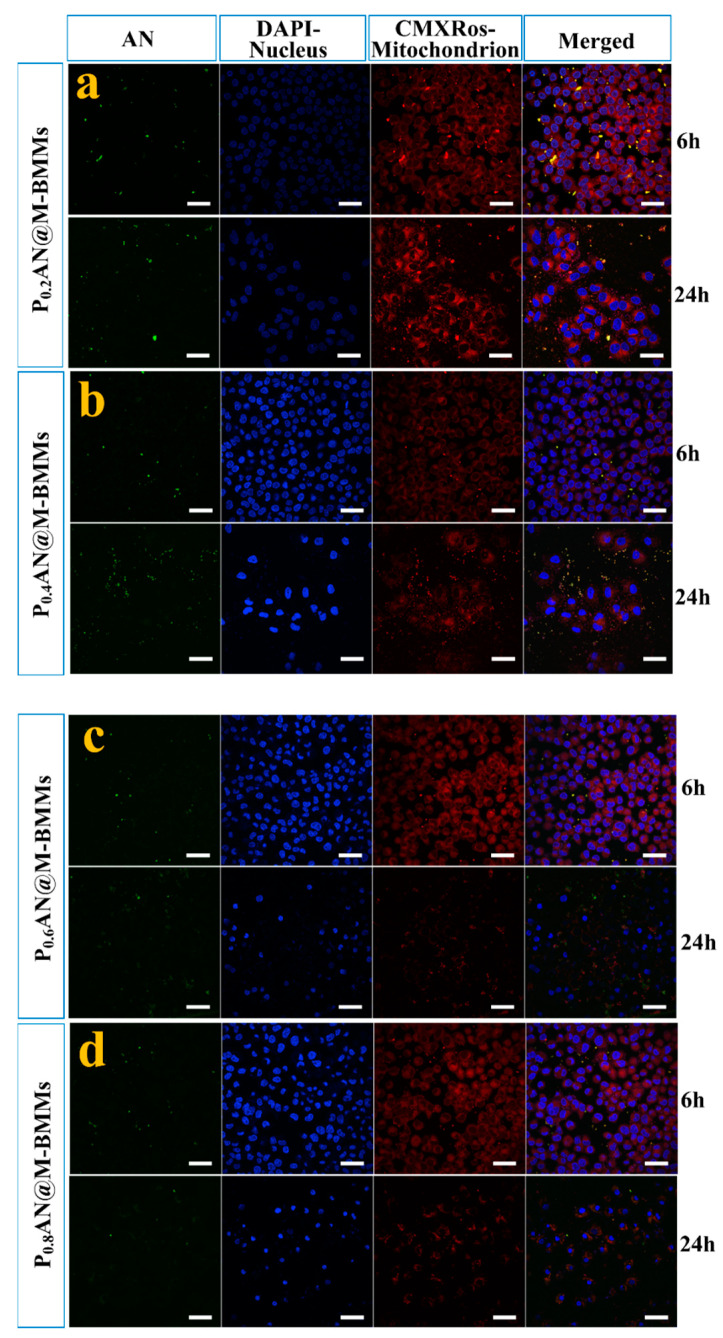
Confocal images of in vitro cellular uptake of PAN@M-BMMs (**a**–**d**) after incubation of 6~24 h in the HeLa cell line. In which the green, red, and blue regions represent the fluorescent AN, cytopainter stained Mitochondrion, and nucleus stained with DAPI, respectively. Scale bars were 50 μm.

**Figure 6:**
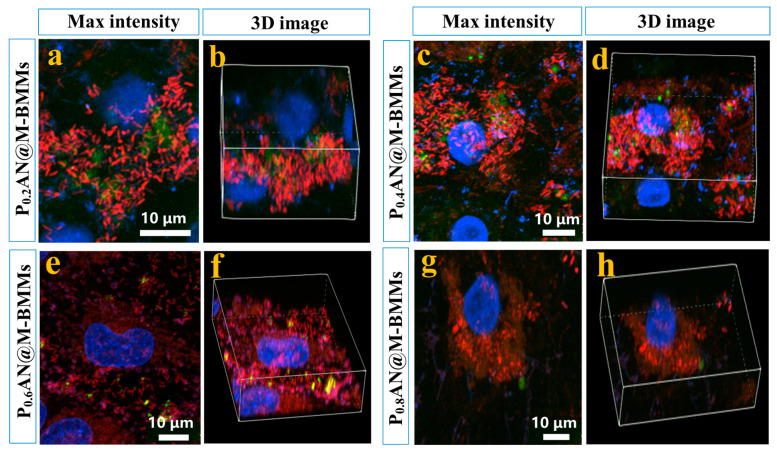
In vitro cellular uptake representation by section of the cell and maximum intensity projection after 24 h incubation in the HeLa cell line. P_0.2_AN@M-BMMs (**a**,**b**), P_0.4_AN@M-BMMs (**c**,**d**), P_0.6_AN@M-BMMs (**e**,**f**), and P_0.8_AN@M-BMMs (**g**,**h**). In which the green, red, and blue regions represent the fluorescent AN, cytopainter stained Mitochondrion, and nucleus stained with DAPI, respectively.

The cellular uptake of the PAN@M-BMMs was preliminarily explored for achieving the therapeutic effects in the target area. As shown in Figure 5, the cellular uptake of the PAN@M-BMMs was assessed using confocal microscopy after incubation for 6 and 24 h. Figure 6 illustrates their intracellular distribution after 24 h of incubation, in which the cross-sectional and maximum intensity projection of the PAN@M-BMMs intake for each cell was depicted using Image Viewer software (NIS-Elements Viewer 5.21.00). As can be seen, the PAN@M-BMMs with various coated-PAN amounts were clearly taken up into the cytoplasm or cell membranes.

**Figure 7:**
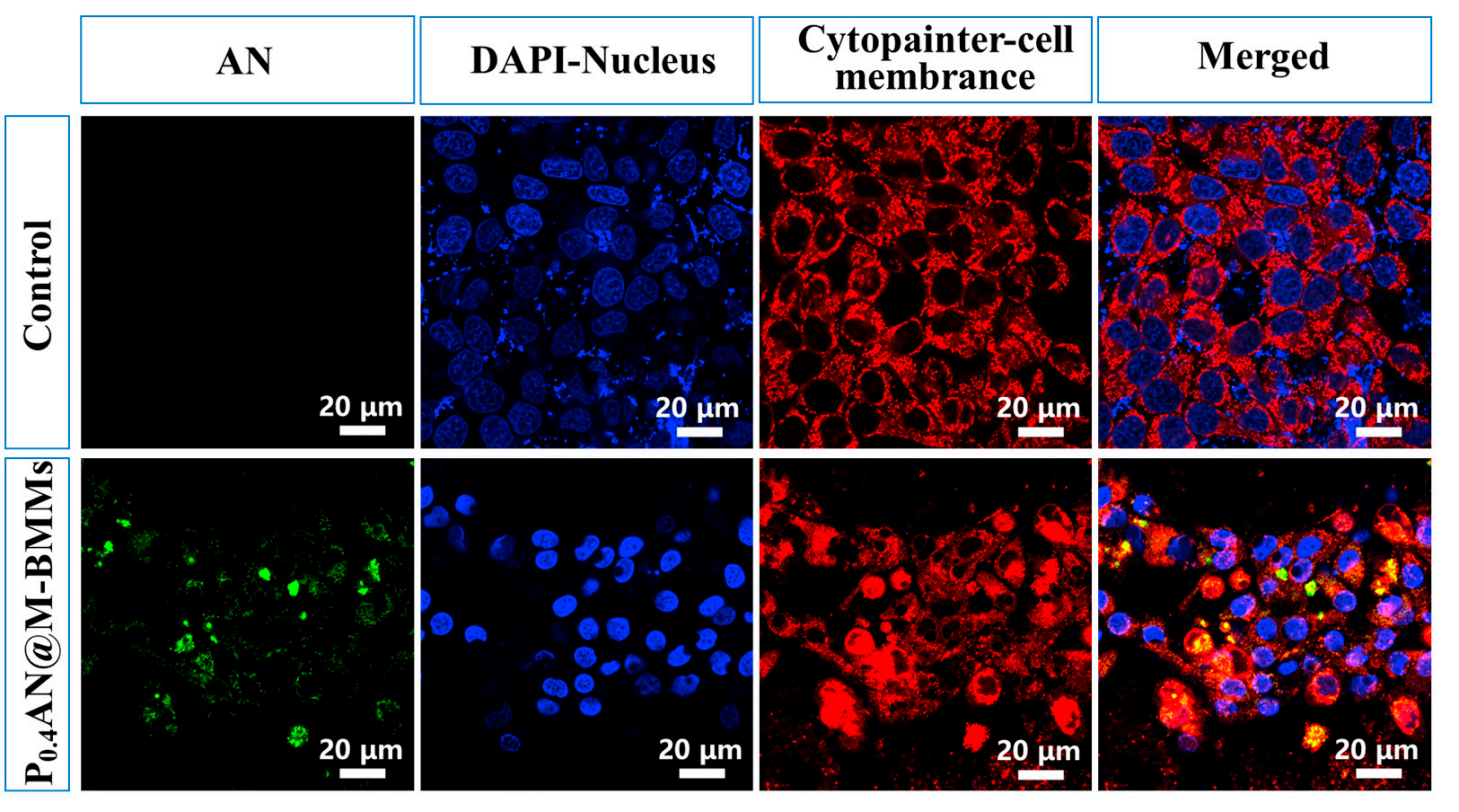
Confocal images of in vitro cellular uptake of P_0.4_AN@M-BMMs after incubation for 48 h in the HeLa cell line and Control cells. In which the green, red, and blue regions represent the fluorescent AN, cytopainter stained Mitochondrion, and nucleus stained with DAPI, respectively.

Taking P_0.4_AN@M-BMMs as an example, Figure 7 provides its detailed observation of entering the HeLa cell line after incubation for 48 h, showing its gradual uptake observed in the confocal images from 6 to 48 h. The experimental results indicated that its cellular uptake process was time-dependent. Similar results of P_0.2_AN@M-BMMs for 48 h were also presented in Figure S13. As proposed by Rennick et al. [56], we speculated that the endocytosis mechanism is one of the main uptake pathways, such as clathrin-mediated endocytosis, fast endophilin-mediated endocytosis, or clathrin-independent carrier/glycosylphosphatidylinositol-anchored protein-enriched early endocytic, which needs to be further investigated.

The fluorescent intensities of the characteristic peak of the doped AN for cells treated by PAN@M-BMMs coated with varying amounts of the PAN shell and control cells were demonstrated via flow cytometry analysis. Each sample was tested at a concentration of 250 µg/mL, respectively.

**Figure 8:**
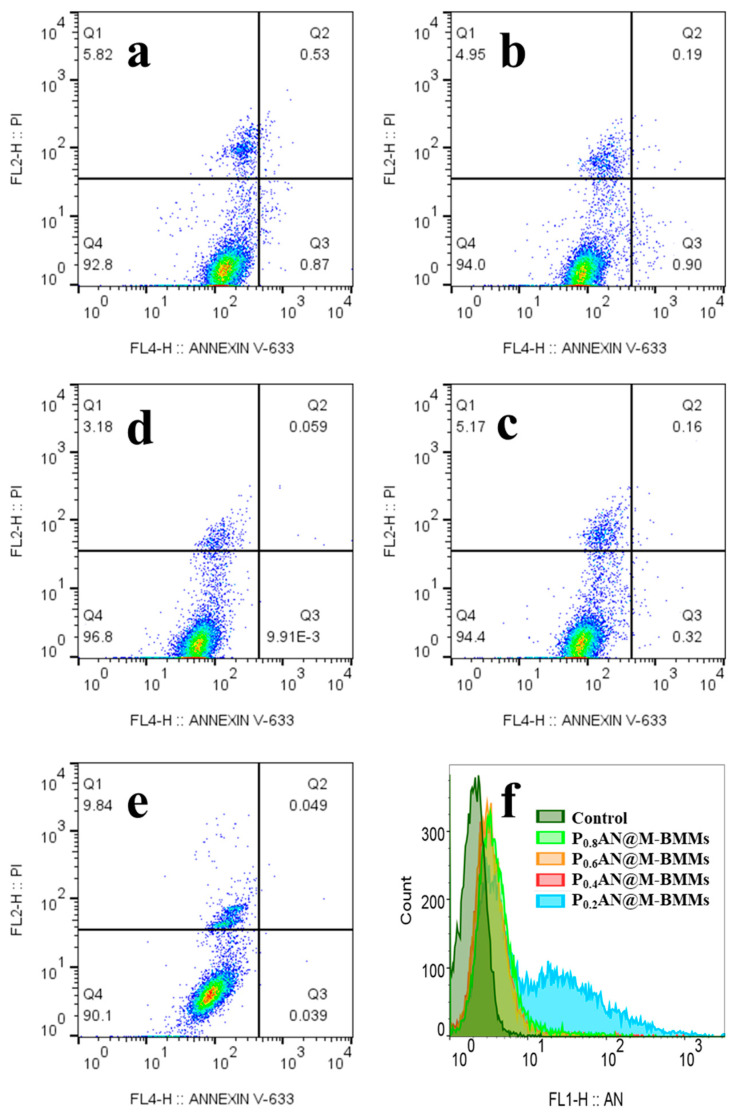
In vitro cell apoptosis after incubation for 24 h in the HeLa cell line. P_0.2_AN@M-BMMs (**a**), P_0.4_AN@M-BMMs (**b**), P_0.6_AN@M-BMMs (**c**), P_0.8_AN@M-BMMs (**d**), control cells (**e**), and the fluorescence intensity after cell uptake measured by flow cytometry (**f**).

Figure 8 from a to e depicts the apoptotic state of cells incubated with various PAN@M-BMMs for 24 h. As can be seen, the HeLa cells treated by various P_x_AN@M-BMMs exhibited the similar apoptosis rates, compared to control cells after culture for 24 h. These observations further supported the low cytotoxicity of various P_x_AN@M-BMMs.

As shown in Figure 8f, a rightward shift of the fluorescent peak in the HeLa cell line was observed for each sample, compared with the control cell. In detail, the most significant shift appeared in P_0.2_AN@M-BMMs, followed by P_0.4_AN@M-BMMs, P_0.6_AN@M-BMMs, and P_0.8_AN@M-BMMs. These phenomena might be related to the smaller particle size of P_0.2_AN@M-BMMs (179 nm), which is conducive to facilitating cellular uptake.

### 3.7. Cellular Internalization

**Figure 9:**
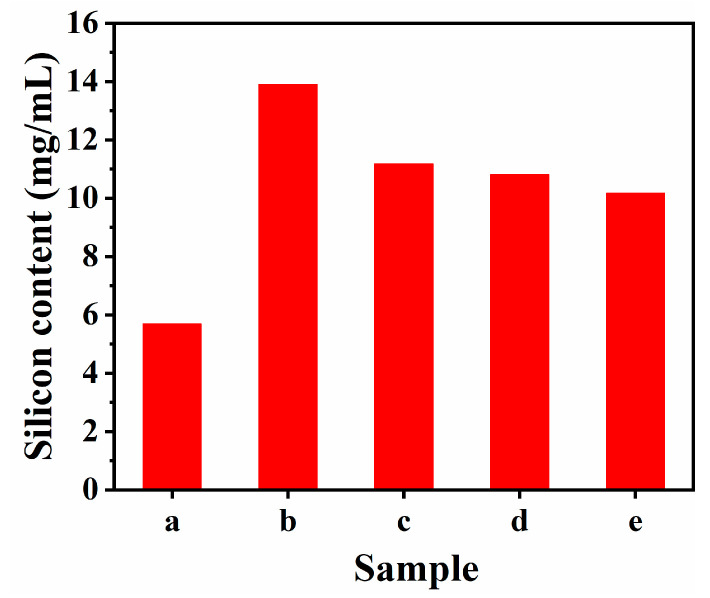
Cellular internalization after incubation for 24 h in the HeLa cell line. Control (a), P_0.2_AN@M-BMMs (b), P_0.4_AN@M-BMMs (c), P_0.6_AN@M-BMMs (d), and P_0.8_AN@M-BMMs (e).

The internalization of fluorescent hybrid materials with varying amounts of PAN encapsulation was assessed using ICP analysis. HeLa cell lines were treated using an experimental method, and the silicon content within the cells was quantified. As can be seen in Figure 9, the ICP analysis corroborated the previous findings, demonstrating that P_0.2_AN@M-BMMs with a lower amount of PAN encapsulation exhibited an enhanced uptake efficiency, especially as evidenced by the highest silicon content observed in the tests (Figure 9b).

### 3.8. Hemocompatibility Test of P_X_AN@M-BMMs

The biocompatibility of drug carriers in blood was evaluated by assessing the hemolysis rate of red blood cells incubated with fluorescent hybrid materials at various concentrations for 1 h. The maximum absorption wavelength of hemoglobin, determined using a UV–Vis spectrophotometer, was found to be 576 nm, which could be selected to measure the hemolytic activity of P_X_AN@M-BMMs.

**Figure 10:**
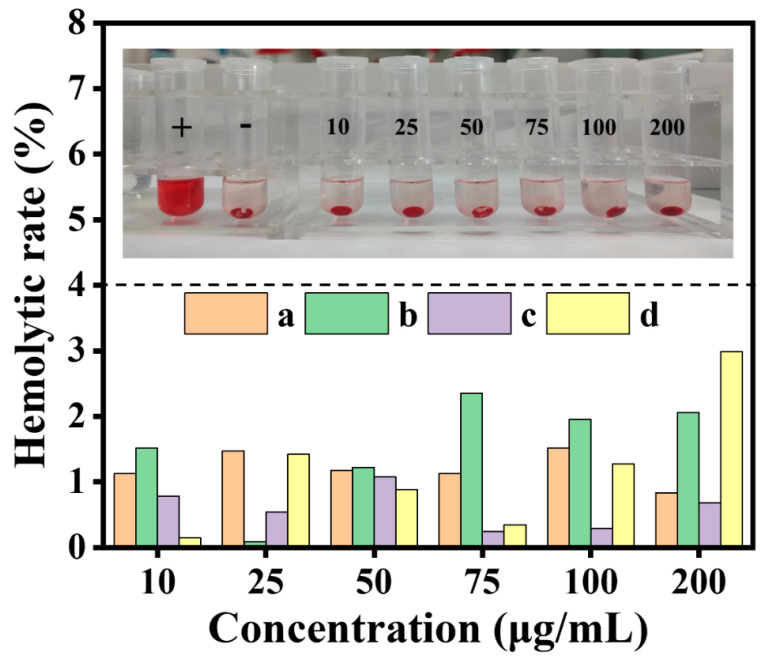
Hemolysis graph and hemolysis rate of P_0.2_AN@M-BMMs (a), P_0.4_AN@M-BMMs (b), P_0.6_AN@M-BMMs (c), and P_0.8_AN@M-BMMs (d). In which the symbols +, − and the numbers represent the positive control, the negative control, and the sample concentration, respectively.

As depicted in Figure 10, the solution in the positive control group displayed a red color, indicating complete hemolysis, which was attributed to the lower osmotic pressure of distilled water compared to that of red blood cells, leading to cell lysis due to water absorption. In contrast, no hemolysis was observed in the isotonic solution of the negative control group. At all tested concentrations, the hemolysis rate was observed to be less than 5%, signifying that the P_X_AN@M-BMMs as a drug carrier possess commendable biocompatibility. These results are consistent with the cell viability in Figure 4, further demonstrating the low cytotoxicity of various P_X_AN@M-BMMs.

## 4. Conclusions

The core–shell structured PAN@M-BMMs were successfully synthesized by modulating the coated-PAN shell thicknesses, and their bimodal mesoporous structural features were further demonstrated; Specially, their fractal structure evolutions deriving from SAXS data indicated an increase in *D_m_* values ranging from 2.74 to 2.87 with the increase in the coated-PAN amount, along with the increased thickness of the coated-PAN shell from 7.7 to 10.5 nm, demonstrating the densification tendency of the coated-PAN shell in PAN@M-BMMs. The obtained PAN@M-BMMs presented a high drug-loading capacity (13.8%) and prominent pH-/temperature-sensitive release characteristics (83.1% at pH 7.4, 25 °C and 17.9% at pH 2, 37 °C). Various characterizations, such as in vitro cell assays, confocal imaging, flow cytometry, and ICP analysis, elucidated that the resultant PAN@M-BMMs exhibited a low cytotoxicity at concentrations up to 100 µg/mL and facilitated cell uptake internalization in a time-dependent manner, in which P_0.2_AN@M-BMMs presented a superior cellular uptake and fluorescent performance, compared to P_0.4_AN@M-BMMs, P_0.6_AN@M-BMMs, and P_0.8_AN@M-BMMs. These phenomena might be related to the smaller particle size of P_0.2_AN@M-BMMs (179 nm), which is conducive to facilitating cellular uptake. The hemolysis rate of below 5% indicated that the PAN@M-BMMs presented a promising prospect for biomedical applications. These findings provide the clinical support for the application of organic–inorganic hybrid mesoporous silica nanomaterials in drug delivery.

## Data Availability

Data will be made available on request.

## Supplementary Materials

The following supporting information can be downloaded at: https://www.mdpi.com/article/10.3390/pharmaceutics17020206/s1, Figure S1. Regression equations and the standard curves of the released-IBU concentration in PBS at (A) pH 2.0/37 °C and (B) pH 7.4/25 °C. Figure S2. FT-IR spectra of BMMs (a), M-BMMs (b), P(NIPAM-co-AA) (c), AN (d), and P_0.8_AN@M-BMMs (e). Figure S3. Particle size distribution of BMMs (a), M-BMMs (b), P_0.2_AN@M-BMMs (c), P_0.4_AN@M-BMMs (d), P_0.6_AN@M-BMMs (e), and P_0.8_AN@M-BMMs (f). Figure S4. TG curves of (a) BMMs, (b) M-BMMs, (c) P_0.2_AN@M-BMMs, (d) P_0.4_AN@M-BMMs, (e) P_0.6_AN@M-BMMs, and (f) P_0.8_AN@M-BMMs. Figure S5. Fluorescence emission spectra of P_0.2_AN@M-BMMs (a), P_0.4_AN@M-BMMs (b), P_0.6_AN@M-BMMs (c), and P_0.8_AN@M-BMMs (d). Figure S6. Size distribution profiles of (A) P_0.2_AN@M-BMMs, (B) P_0.4_AN@M-BMMs, (C) P_0.6_AN@M-BMMs, and (D) P_0.8_AN@M-BMMs, (a) pH 2.0/37 °C, (b) pH 7.4/37 °C, (c) pH 2.0/25 °C, and (d) pH 7.4/25 °C. Figure S7. Hydrodynamic diameter illustrations of (a) P_0.2_AN@M-BMMs, (b) P_0.4_AN@M-BMMs, (c) P_0.6_AN@M-BMMs, and (d) P_0.8_AN@M-BMMs. Figure S8. Cumulative release percent of IBU under pH 2.0/37 °C (A) and pH 7.4/25 °C (B), (a) I/P_0.2_@BMMs-7, (b) I/P_0.6_@BMMs-7, and (c) I/P_0.8_@BMMs-7. Figure S9. Ln-Ln plots originating from the SAXS patterns (A) and PDDF profiles (B) of P_0.2_AN@M-BMMs during the drug-loading process, (a) 1 h, (b) 3 h, (c) 5 h, (d) 8 h, (e) 12 h, (f) 24 h, and (g) 48 h. Red lines in Figure S9A: the fitting lines based on the power law, the vertical offset values were presented in the right Y-axis. Figure S10. Ln-Ln plots originating from the SAXS patterns and PDDF profiles of P_0.2_AN@M-BMMs during the drug-releasing process at pH 2.0/37 °C (A), (B) and pH 7.4/25 °C (C), (D), (a) 1 h, (b) 3 h, (c) 5 h, (d) 8 h, (e) 10 h, (f) 12 h, and (g) 24 h. Red lines in Figure S10C and Figure S10D: the fitting lines based on the power law, the vertical offset values were presented in the right Y-axis. Figure S11. Representative scheme of the IBU-adsorption energies in PAN@M-BMMs. BMMs (a), PAN@M-BMMs with the polymerization degree of 3 (b) and 5 (c). Figure S12. Interaction illustrations between IBU and surfaces_(101)_ of BMMs core or coated-PAN shell. BMMs (a), PAN@BMMs with the polymerization degree of 3 (b) and 5 (c). Isosurface = 0.001. Figure S13. Confocal images of in vitro cellular uptake of P_0.2_AN@M-BMMs after incubation 48 h in the HeLa cell line. In which the green, red, and blue regions represent the fluorescent AN, cytopainter stained Mitochondrion, and nucleus stained with DAPI, respectively. Table S1. Collections of various polymer-loaded amounts for synthetic PAN@M-BMMs. Table S2. Collections of the cumulative IBU-releasing percent from P_0.4_AN@M-BMMs at pH 2.0/37 °C and pH 7.4/25 °C in the releasing solution. Table S3. Collections of the *D_m_* values, linear range, and possible maximum particle size (*D_max_*). Table S4. Collections of the fractal dimension values, linear range, and possible maximum particle size (*D_max_*) of P_0.2_AN@M-BMMs during the drug-loading process. Table S5. Collections of the fractal dimension values, linear range, and possible maximum particle size (*D_max_*) of P_0.2_AN@M-BMMs during the drug-releasing process at pH 2.0/37 °C. Table S6. Collections of the fractal dimension values, linear range, and possible maximum particle size (*D_max_*) of P_0.2_AN@M-BMMs during the drug-releasing process at pH 7.4/25 °C.
