# Supplementary material for: Dual pH- and Temperature-Responsive Performance and Cytotoxicity of N-Isopropylacrylamide and Acrylic Acid Functionalized Bimodal Mesoporous Silicas with Core–Shell Structure and Fluorescent Feature for Hela Cell
Source: Pharmaceutics. 2025 Feb 6;17(2):206. doi: 10.3390/pharmaceutics17020206 (PMC11859581; doi:10.3390/pharmaceutics17020206)
Supplement: Supplementary file 1 [file pharmaceutics-17-00206-s001.zip › pharmaceutics-3382674-supplementary.pdf]

Dual pH- and Temperature-Responsive Performance and Cytotoxicity of N-Isopropylacrylamide and Acrylic Acid Functionalized Bimodal Mesoporous Silicas with Core–Shell Structure and Fluorescent Feature for HeLa Cell

Huijie Ge [1], Xiaoli Wang [1], Shiyang Bai [1], Yuhua Bi [1], Fei Liu [1], Jihong Sun [1,*,†], Wenliang Fu [2,*,†] and Donggang Xu [2]

[1] Beijing Key Laboratory for Green Catalysis and Separation, Institute of Matter Science, Beijing University of Technology, Beijing 100124, China

[2] Beijing Institute of Basic Medical Sciences, Beijing 100850, China

Electronic Supplementary Information

Figure S1. Regression equations and the standard curves of the released-IBU concentration in PBS at (A) pH 2.0/37 °C and (B) pH 7.4/25 °C.

Figure S2. FT-IR spectra of BMMs (a), M-BMMs (b), P(NIPAM-co-AA) (c), AN (d), and $P_{0.8}$AN@M-BMMs (e).

Figure S3. Particle size distribution of BMMs (a), M-BMMs (b), $P_{0.2}$AN@M-BMMs (c), $P_{0.4}$AN@M-BMMs (d), $P_{0.6}$AN@M-BMMs (e), and $P_{0.8}$AN@M-BMMs (f).

Figure S4. TG curves of (a) BMMs, (b) M-BMMs, (c) $P_{0.2}$AN@M-BMMs, (d) $P_{0.4}$AN@M-BMMs, (e) $P_{0.6}$AN@M-BMMs, and (f) $P_{0.8}$AN@M-BMMs.

Figure S5. Fluorescence emission spectra of $P_{0.2}$AN@M-BMMs (a), $P_{0.4}$AN@M-BMMs (b), $P_{0.6}$AN@M-BMMs (c), and $P_{0.8}$AN@M-BMMs (d).

Figure S6. Size distribution profiles of (A) $P_{0.2}$AN@M-BMMs, (B) $P_{0.4}$AN@M-BMMs, (C) $P_{0.6}$AN@M-BMMs, and (D) $P_{0.8}$AN@M-BMMs, (a) pH 2.0/37 °C, (b) pH 7.4/37 °C, (c) pH 2.0/25 °C, and (d) pH 7.4/25 °C.

Figure S7. Hydrodynamic diameter illustrations of (a) $P_{0.2}$AN@M-BMMs, (b) $P_{0.4}$AN@M-BMMs, (c) $P_{0.6}$AN@M-BMMs, and (d) $P_{0.8}$AN@M-BMMs.

Figure S8. Cumulative release percent of IBU under pH 2.0/37 °C (A) and pH 7.4/25 °C (B), (a) I/$P_{0.2}$@BMMs-7, (b) I/$P_{0.6}$@BMMs-7, and (c) I/$P_{0.8}$@BMMs-7.

Figure S9. Ln-Ln plots originating from the SAXS patterns (A) and PDDF profiles (B) of $P_{0.2}$AN@M-BMMs during the drug-loading process, (a) 1 h, (b) 3 h, (c) 5 h, (d) 8 h, (e) 12 h, (f) 24 h, and (g) 48 h. Red lines in Figure S9A: the fitting lines based on the power law, the vertical offset values were presented in the right Y-axis.

Figure S10. Ln-Ln plots originating from the SAXS patterns and PDDF profiles of $P_{0.2}$AN@M-BMMs during the drug-releasing process at pH 2.0/37 °C (A), (B) and pH 7.4/25 °C (C), (D), (a) 1 h, (b) 3 h, (c) 5 h, (d) 8 h, (e) 10 h, (f) 12 h, and (g) 24 h. Red lines in Figure S10C and Figure S10D: the fitting lines based on the power law, the vertical offset values were presented in the right Y-axis.

Figure S11. Representative scheme of the IBU-adsorption energies in PAN@M-BMMs. BMMs (a), PAN@M-BMMs with the polymerization degree of 3 (b) and 5 (c).

Figure S12. Interaction illustrations between IBU and surfaces$_{(101)}$ of BMMs core or coated-PAN shell. BMMs (a), PAN@BMMs with the polymerization degree of 3 (b) and 5 (c). Isosurface = 0.001.

Figure S13. Confocal images of in vitro cellular uptake of $P_{0.2}$AN@M-BMMs after incubation 48 h in the HeLa cell line. In which, the green, red, and blue regions represent the fluorescent AN, cytopainter stained Mitochondrion, and nucleus stained with DAPI, respectively.

Table S1. Collections of various polymer-loaded amounts for synthetic PAN@M-BMMs.

Table S2. Collections of the cumulative IBU-releasing percent from $P_{0.4}$AN@M-BMMs at pH 2.0/37 °C and pH 7.4/25 °C in the releasing solution.

Table S3. Collections of the D_m values, linear range, and possible maximum particle size (D_{max}).

Table S4. Collections of the fractal dimension values, linear range, and possible maximum particle size (D_{max}) of $P_{0.2}$AN@M-BMMs during the drug-loading process.

Table S5. Collections of the fractal dimension values, linear range, and possible maximum particle size (D_{max}) of $P_{0.2}$AN@M-BMMs during the drug-releasing process at pH 2.0/37 °C.

Table S6. Collections of the fractal dimension values, linear range, and possible maximum particle size (D_{max}) of $P_{0.2}$AN@M-BMMs during the drug-releasing at pH 7.4/25 °C.

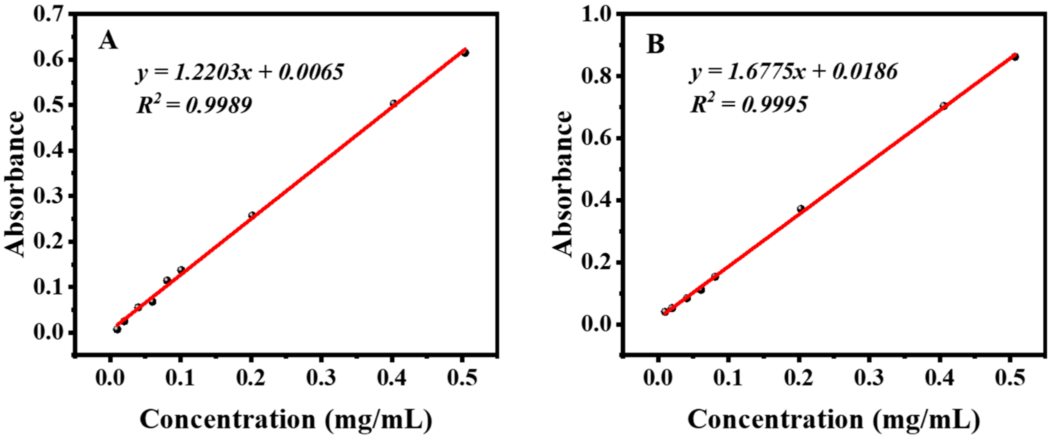

Figure S1. Regression equations and the standard curves of the released-IBU concentration in PBS at (A) pH 2.0/37 °C and (B) pH 7.4/25 °C.

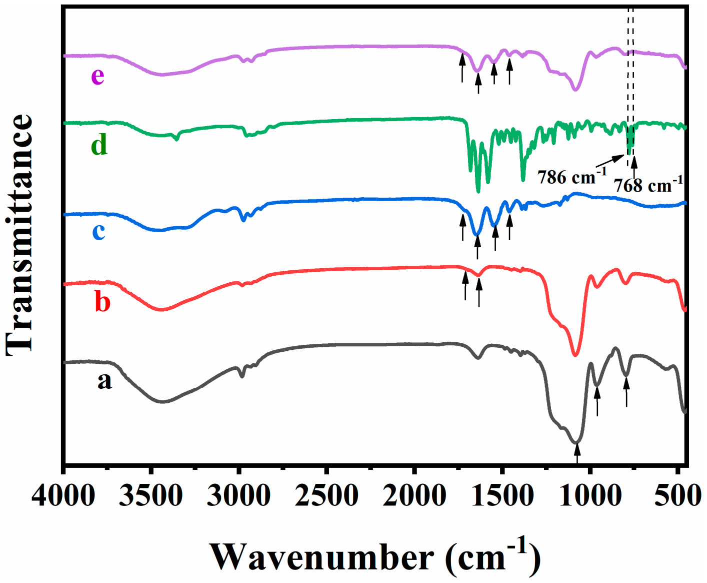

Figure S2. FT-IR spectra of BMMs (a), M-BMMs (b), P(NIPAM-co-AA) (c), AN (d), and $P_{0.8}$AN@M-BMMs (e).

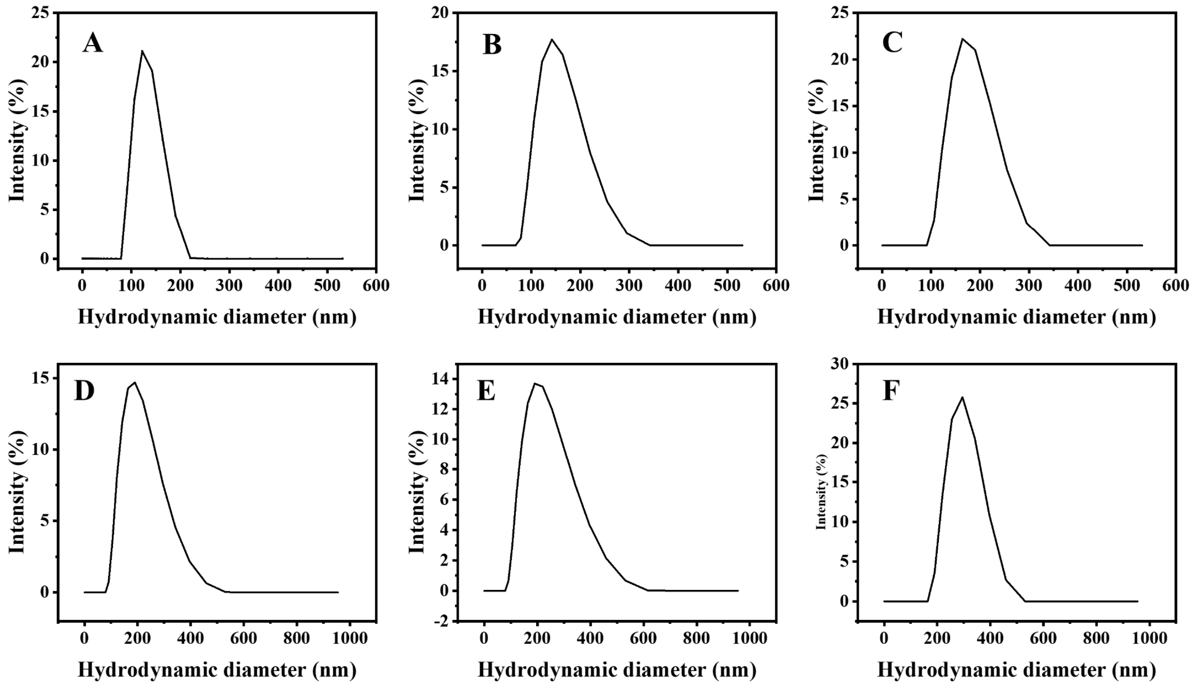

Figure S3. Particle size distribution of BMMs (a), M-BMMs (b), $P_{0.2}$AN@M-BMMs (c), $P_{0.4}$AN@M-BMMs (d), $P_{0.6}$AN@M-BMMs (e), and $P_{0.8}$AN@M-BMMs (f).

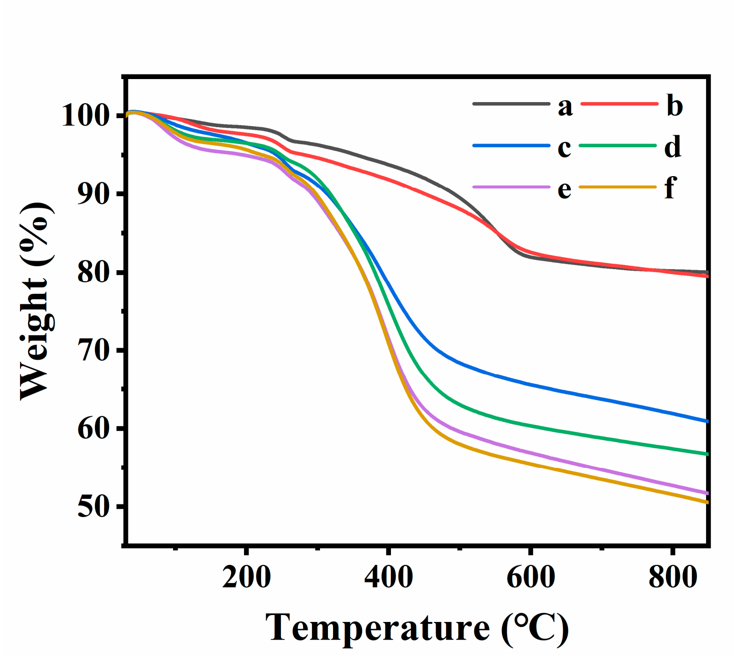

Figure S4. TG curves of (a) BMMs, (b) M-BMMs, (c) $P_{0.2}$AN@M-BMMs, (d) $P_{0.4}$AN@M-BMMs, (e) $P_{0.6}$AN@M-BMMs, and (f) $P_{0.8}$AN@M-BMMs.

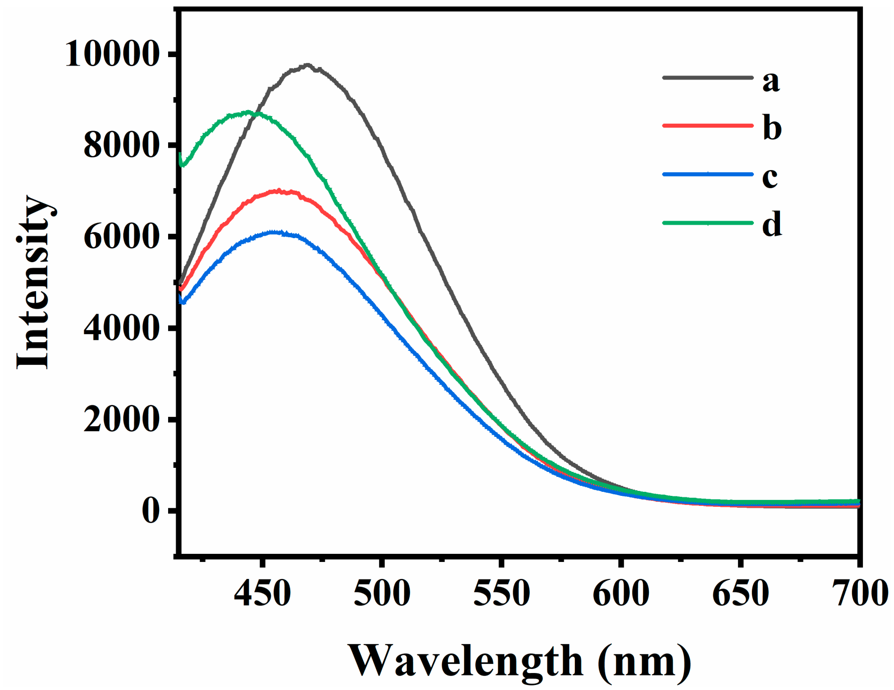

Figure S5. Fluorescence emission spectra of $P_{0.2}$AN@M-BMMs (a), $P_{0.4}$AN@M-BMMs (b), $P_{0.6}$AN@M-BMMs (c), and $P_{0.8}$AN@M-BMMs (d).

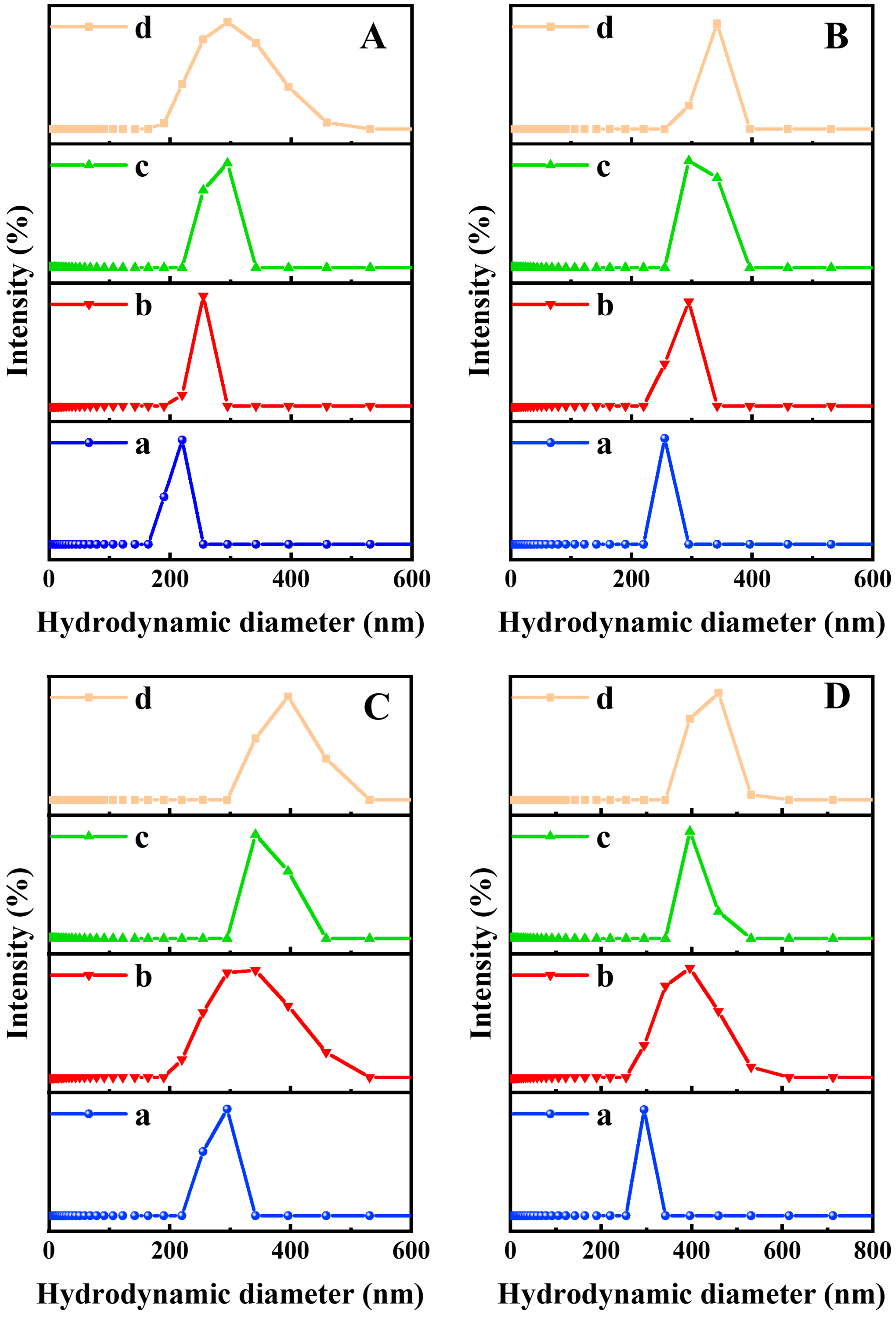

Figure S6. Size distribution profiles of (A) $P_{0.2}$AN@M-BMMs, (B) $P_{0.4}$AN@M-BMMs, (C) $P_{0.6}$AN@M-BMMs, and (D) $P_{0.8}$AN@M-BMMs, (a) pH 2.0/37 °C, (b) pH 7.4/37 °C, (c) pH 2.0/25 °C, and (d) pH 7.4/25 °C.

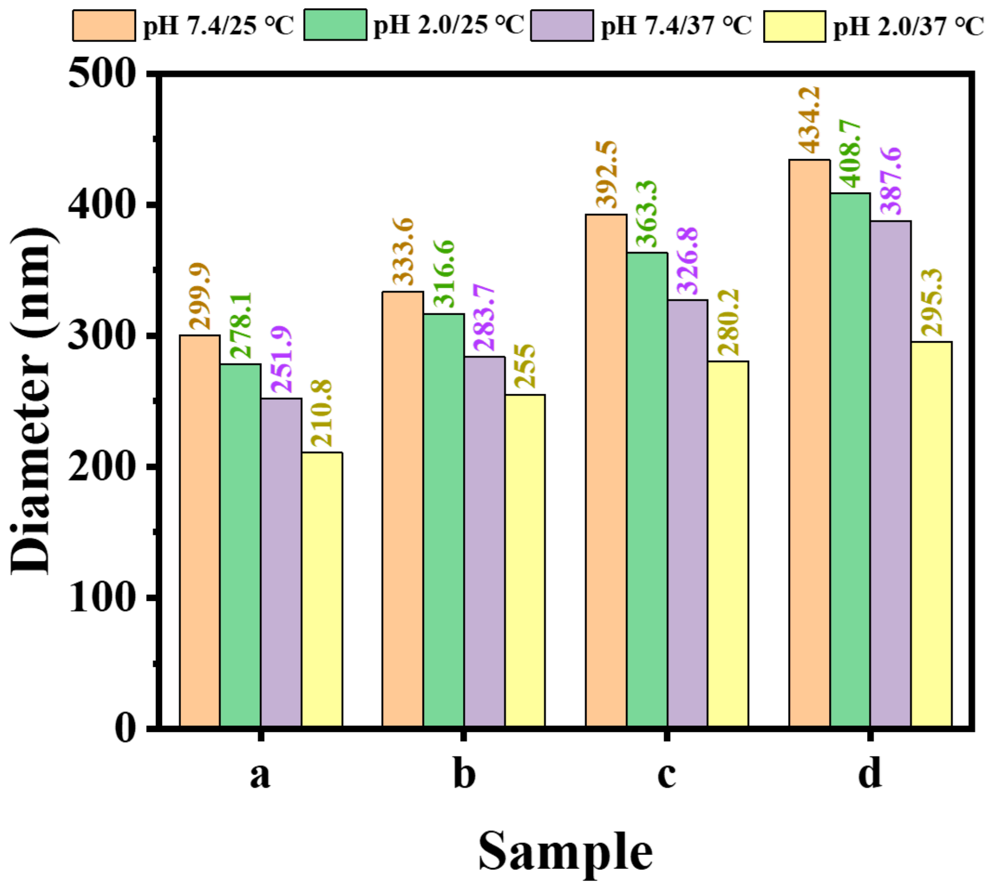

Figure S7. Hydrodynamic diameter illustrations of (a) $P_{0.2}$AN@M-BMMs, (b) $P_{0.4}$AN@M-BMMs, (c) $P_{0.6}$AN@M-BMMs, and (d) $P_{0.8}$AN@M-BMMs.

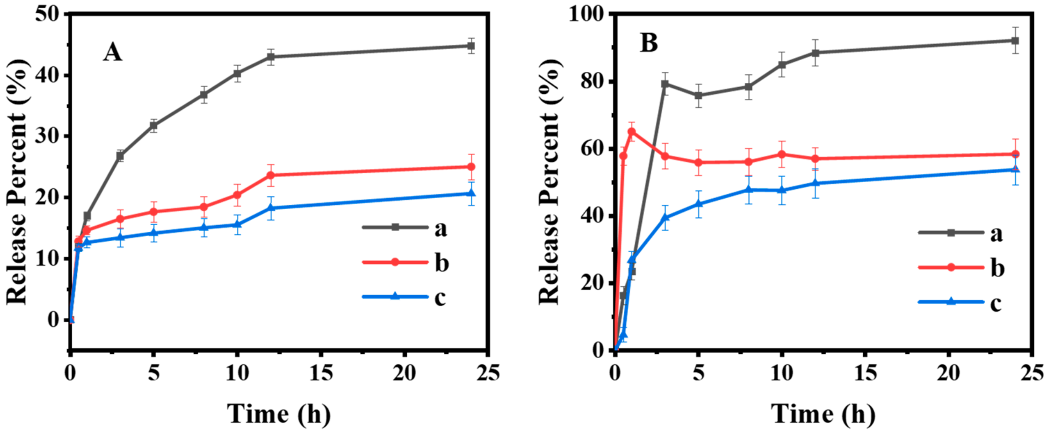

Figure S8. Cumulative release percent of IBU under pH 2.0/37 °C (A) and pH 7.4/25 °C (B), (a) I/$P_{0.2}$@BMMs-7, (b) I/$P_{0.6}$@BMMs-7, and (c) I/$P_{0.8}$@BMMs-7.

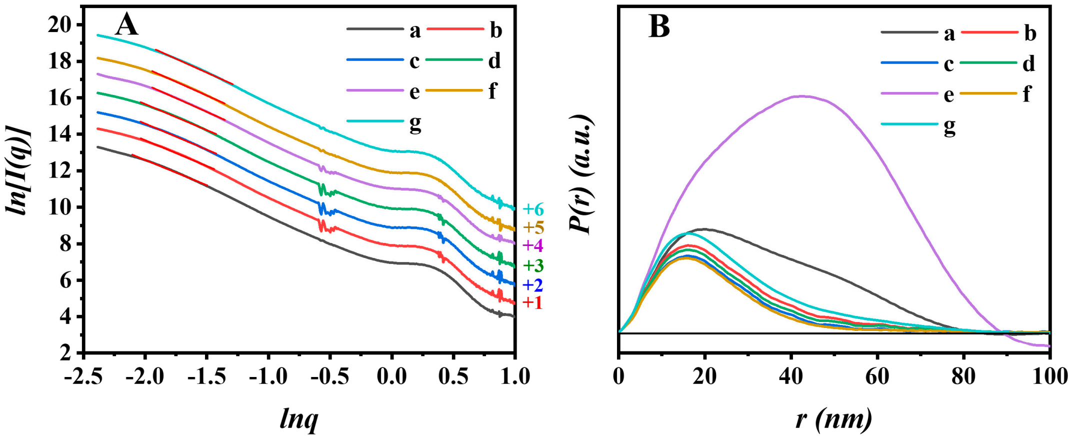

Figure S9. Ln-Ln plots originating from the SAXS patterns (A) and PDDF profiles (B) of $P_{0.2}$AN@M-BMMs during the drug-loading process, (a) 1 h, (b) 3 h, (c) 5 h, (d) 8 h, (e) 12 h, (f) 24 h, and (g) 48 h. Red lines in Figure S9A: the fitting lines based on the power law, the vertical offset values were presented in the right Y-axis.

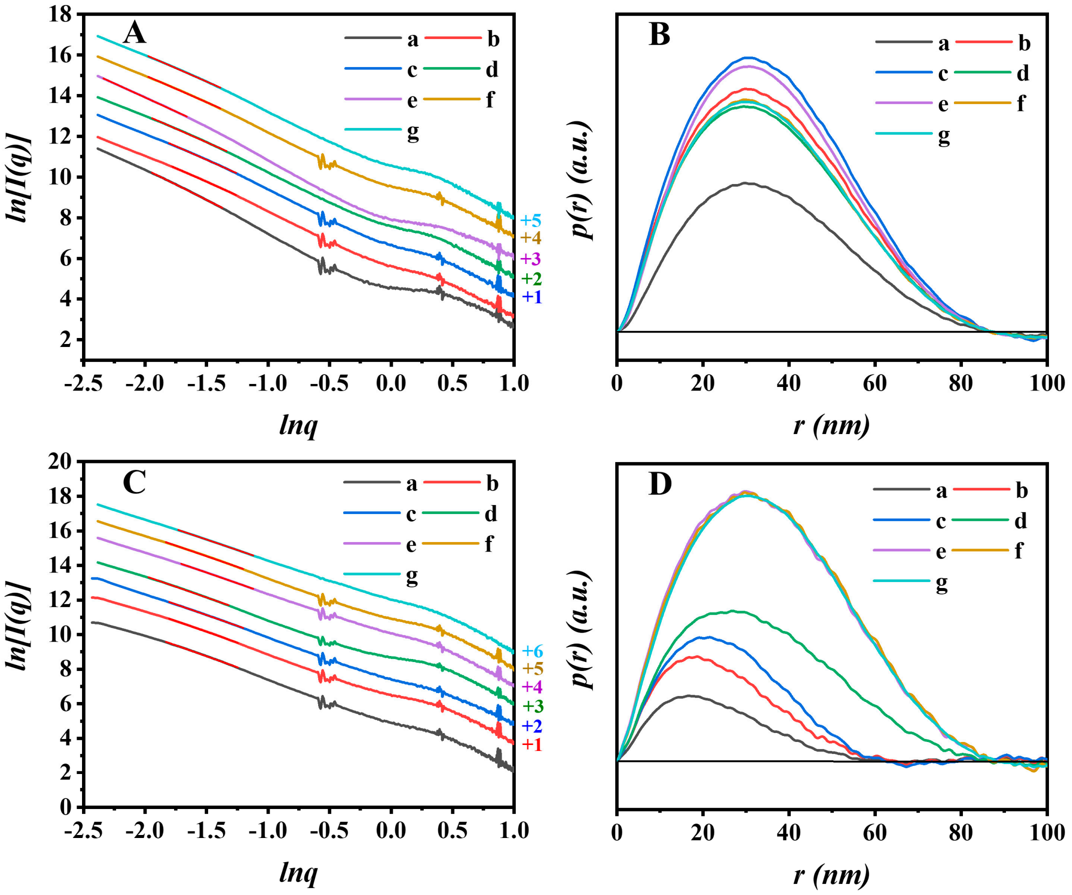

Figure S10. Ln-Ln plots originating from the SAXS patterns and PDDF profiles of $P_{0.2}$AN@M-BMMs during the drug-releasing process at pH 2.0/37 °C (A), (B) and pH 7.4/25 °C (C), (D), (a) 1 h, (b) 3 h, (c) 5 h, (d) 8 h, (e) 10 h, (f) 12 h, and (g) 24 h. Red lines in Figure S10C and Figure S10D : the fitting lines based on the power law, the vertical offset values were presented in the right Y-axis.

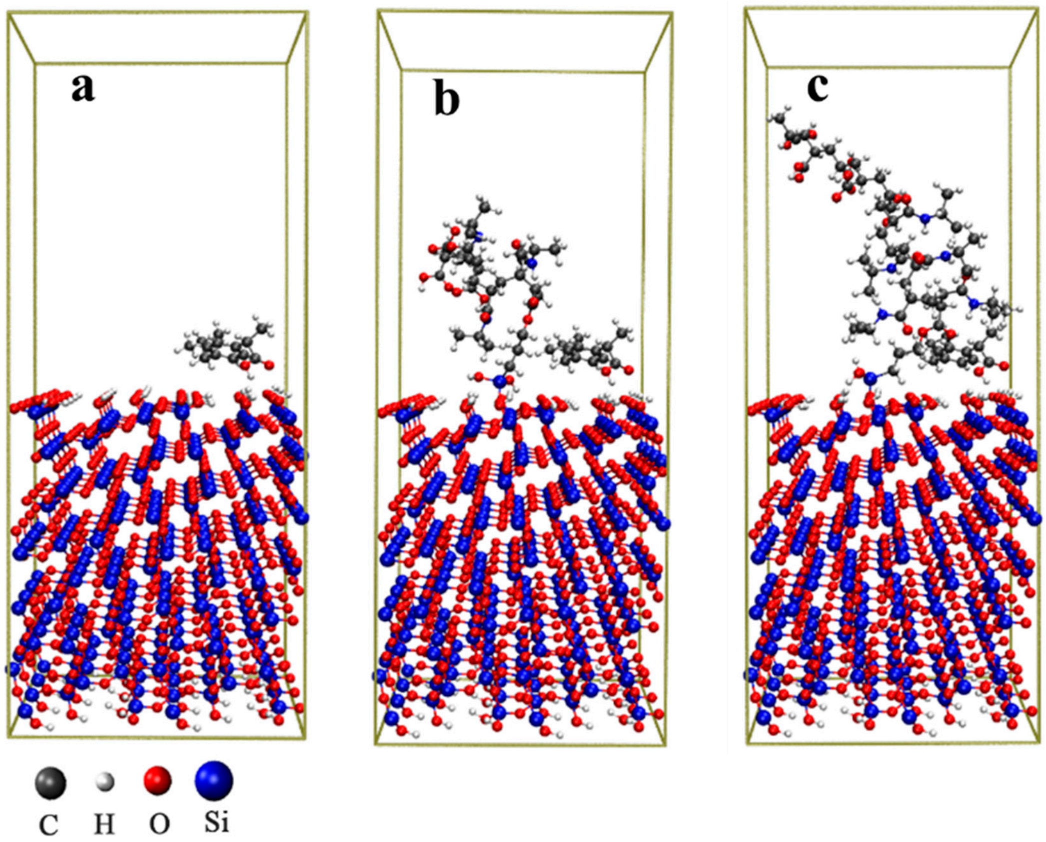

Figure S11. Representative scheme of the IBU-adsorption energies in PAN@M-BMMs. BMMs (a), PAN@M-BMMs with the polymerization degree of 3 (b) and 5 (c).

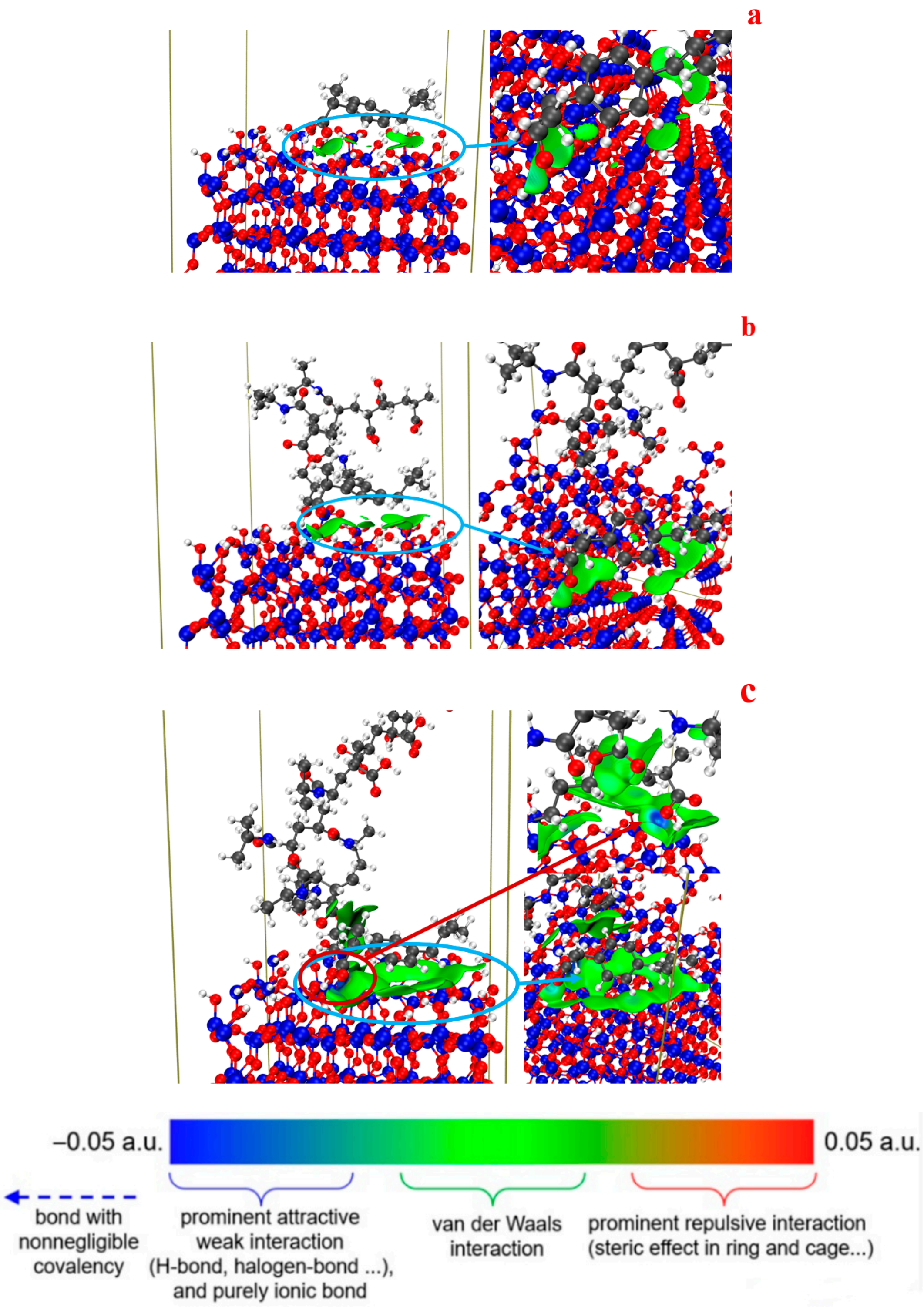

Figure S12. Interaction illustrations between IBU and surfaces$_{(101)}$ of BMMs core or coated-PAN shell. BMMs (a), PAN@BMMs with the polymerization degree of 3 (b) and 5 (c). Isosurface = 0.001.

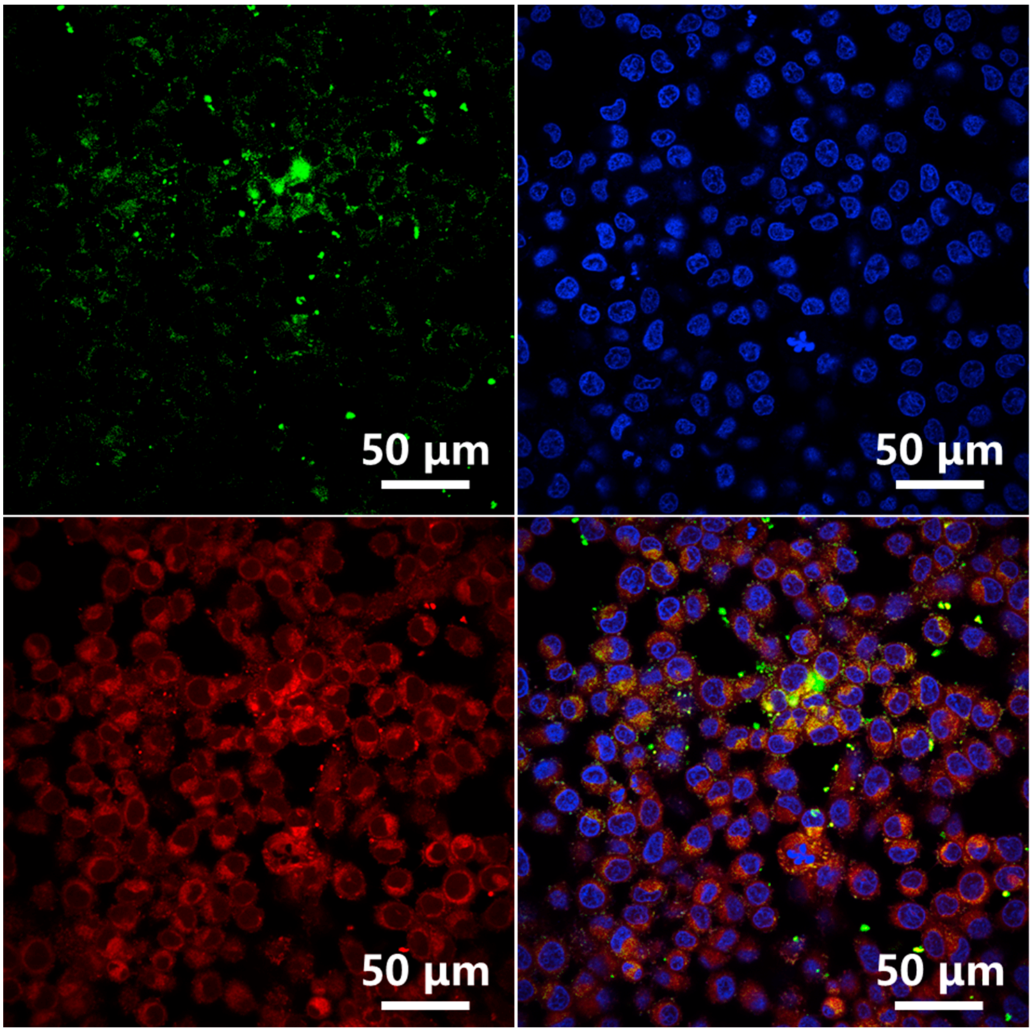

Figure S13. Confocal images of in vitro cellular uptake of $P_{0.2}$AN@M-BMMs after incubation 48 h in the HeLa cell line. In which, the green, red, and blue regions represent the fluorescent AN, cytopainter stained Mitochondrion, and nucleus stained with DAPI, respectively.

Table S1. Collections of various polymer-loaded amounts for synthetic PAN@M-BMMs.

Samples	M-t-BMMs (mg)	NIPAM (mg)	AA(μL)	BIS (mg)
$P_{0.2}$AN@M-BMMs	200	40	2.8	7.3
$P_{0.4}$AN@M-BMMs	200	80	5.6	14.6
$P_{0.6}$AN@M-BMMs	200	120	8.4	21.9
$P_{0.8}$AN@ M-BMMs	200	160	11.2	29.2

Table S2. Collections of the cumulative IBU-releasing percent from $P_{0.4}$AN@M-BMMs at pH 2.0/37 °C and pH 7.4/25 °C in the releasing solution.

Time (h)	Cumulative Release Percent (%)	
	pH 2/37℃	pH 7.4/25℃
0.5	11.70	72.67
1	14.46	67.24
1.5	16.48	77.27
2	15.22	81.02
3	15.77	75.74
6	17.14	82.27
8	17.93	75.50
10	17.68	79.61
12	17.40	95.34
24	17.91	83.10

Table S3. Collections of the D_m values, linear range, and possible maximum particle size.

Samples	D_m value	Linear range	D_{max} (nm)	PAN-coated thickness (nm)
BMMs	2.70	-2.18 ~ -1.89	53.4	-
M-BMMs	2.71	-2.18 ~ -1.78	54.4	-
$P_{0.2}$AN@M-BMMs	2.74	-2.01 ~ -1.54	76.1	10.8
$P_{0.4}$AN@M-BMMs	2.83	-2.07 ~ -1.66	79.1	7.7
$P_{0.6}$AN@M-BMMs	2.85	-2.04 ~ -1.54	82.7	12.5
$P_{0.8}$AN@M-BMMs	2.87	-2.10 ~ -1.54	84.5	10.5

Table S4. Collections of the fractal dimension values, linear range, and possible maximum particle size of $P_{0.2}$AN@M-BMMs during the drug-loading process.

Time (h)	Fractal dimension	Linear range	D_{max} (nm)
1	D_m=2.76	$-2.10 < lnq < -1.50$	81.2
3	D_m=2.83	$-2.04 < lnq < -1.44$	82.7
5	D_m=2.85	$-2.04 < lnq < -1.42$	83.0
8	D_m=2.86	$-2.04 < lnq < -1.42$	83.7
12	D_s=2.99	$-1.94 < lnq < -1.35$	88.4
24	D_s=2.98	$-1.94 < lnq < -1.35$	89.2
48	D_s=2.96	$-1.91 < lnq < -1.29$	90.2

Table S5. Collections of the fractal dimension values, linear range, and possible maximum particle size of $P_{0.2}$AN@M-BMMs during the drug-releasing process at pH 2.0/37 °C.

Time（h）	Fractal dimension	Linear range	D_{max} (nm)
1	D_s=2.97	$-1.94<lnq<-1.37$	85.8
3	D_m=2.69	$-1.78<lnq<-1.26$	86.0
5	D_m=2.68	$-1.81<lnq<-1.26$	85.9
8	D_m=2.67	$-1.94<lnq<-1.34$	86.1
10	D_m =2.76	$-2.34 <lnq<-1.66$	86.3
12	D_m =2.66	$-1.97 <lnq<-1.34$	86.2
24	D_m =2.64	$-1.97<lnq<-1.39$	86.1

Table S6. Collections of the fractal dimension values, linear range, and possible maximum particle size of $P_{0.2}$AN@M-BMMs during the drug-releasing process at pH 7.4/25 °C.

Time（h）	Fractal dimension	Linear range	D_{max} (nm)
1	D_m=2.53	$-1.94<lnq<-1.37$	57.5
3	D_m=2.50	$-1.78<lnq<-1.26$	59.3
5	D_m=2.48	$-1.81<lnq<-1.26$	62.1
8	D_m=2.48	$-1.94<lnq<-1.34$	84.2
10	D_m =2.47	$-2.34 <lnq<-1.66$	86.5
12	D_m =2.46	$-1.97 <lnq<-1.34$	86.9
24	D_m =2.42	$-1.97<lnq<-1.39$	87.8
